# Candida albicans Enhances the Progression of Oral Squamous Cell Carcinoma *In Vitro* and *In Vivo*

**DOI:** 10.1128/mBio.03144-21

**Published:** 2022-01-04

**Authors:** Máté Vadovics, Jemima Ho, Nóra Igaz, Róbert Alföldi, Dávid Rakk, Éva Veres, Balázs Szücs, Márton Horváth, Renáta Tóth, Attila Szücs, Andrea Csibi, Péter Horváth, László Tiszlavicz, Csaba Vágvölgyi, Joshua D. Nosanchuk, András Szekeres, Mónika Kiricsi, Rhonda Henley-Smith, David L. Moyes, Selvam Thavaraj, Rhys Brown, László G. Puskás, Julian R. Naglik, Attila Gácser

**Affiliations:** a Department of Microbiology, University of Szeged, Szeged, Hungary; b Doctoral School of Biology, University of Szeged, Szeged, Hungary; c Department of Biochemistry and Molecular Biology, University of Szeged, Szeged, Hungary; d AstridBio Technologies Ltd., Szeged, Hungary; e Synthetic and System Biology Unit, Biological Research Centre (BRC), Szeged, Hungary; f Department of Pathology, University of Szeged, Szeged, Hungary; g Division of Infectious Diseases, Department of Medicine, Albert Einstein College of Medicine, Bronx, New York, USA; h Department of Microbiology and Immunology, Albert Einstein College of Medicine, Bronx, New York, USA; i Centre for Host-Microbiome Interactions, Faculty of Dentistry, Oral & Craniofacial Sciences, King’s College London, London, United Kingdom; j Centre for Oral, Clinical and Translational Science, Faculty of Dentistry, Oral & Craniofacial Sciences, King’s College London, London, United Kingdom; k King’s Health Partners, Head and Neck Cancer Biobank, Guy's & St Thomas' NHS Foundation Trust, London, United Kingdom; l HCEMM-USZ Fungal Pathogens Research Group, Department of Microbiology, University of Szeged, Szeged, Hungary; m MTA-SZTE Lendület Mycobiome Research Group, University of Szeged, Szeged, Hungary; University of Melbourne; University of Texas Health Science Center

**Keywords:** *Candida albicans*, cancer, oral squamous cell carcinoma, progression

## Abstract

Oral squamous cell carcinoma (OSCC) is associated with oral Candida albicans infection, although it is unclear whether the fungus promotes the genesis and progression of OSCC or whether cancer facilitates fungal growth. In this study, we investigated whether C. albicans can potentiate OSCC tumor development and progression. *In vitro*, the presence of live C. albicans, but not Candida parapsilosis, enhanced the progression of OSCC by stimulating the production of matrix metalloproteinases, oncometabolites, protumor signaling pathways, and overexpression of prognostic marker genes associated with metastatic events. C. albicans also upregulated oncogenes in nonmalignant cells. Using a newly established xenograft *in vivo* mouse model to investigate OSCC-C. albicans interactions, oral candidiasis enhanced the progression of OSCC through inflammation and induced the overexpression of metastatic genes and significant changes in markers of the epithelial-mesenchymal transition. Finally, using the 4-nitroquinoline 1-oxide (4NQO) murine model, we directly correlate these *in vitro* and short-term *in vivo* findings with the progression of oncogenesis over the long term. Taken together, these data indicate that C. albicans upregulates oncogenes, potentiates a premalignant phenotype, and is involved in early and late stages of malignant promotion and progression of oral cancer.

## INTRODUCTION

The head and neck account for 2 to 4% of all cancer cases, which includes neoplasms that affect several regions of the oral cavity, pharyngeal sites, and salivary glands. Approximately 90% of oral neoplasms are squamous cell carcinomas (OSCC) (1). OSCC is the 16th most common cancer worldwide (2) and 6th in the United States (3). In Europe, the incidence of oral cancer is especially high in Central and Eastern Europe, and both morbidity and mortality rates are highest in Hungary (4). Risk factors for oral cancer include poor oral hygiene, tobacco use, alcohol use, and meat consumption (5). OSCC is treated by surgery, radiation, and chemotherapy. Chemotherapy and radiotherapy, when used simultaneously, provide a synergistic benefit against OSCC (6). Currently, the primary treatment mode for OSCC is surgery followed by radiotherapy or chemoradiotherapy depending on risk factors. Adverse effects include mucositis and myelosuppression (7), which also affect the composition, quantity, and complexity of the oral microbiota (8–10).

Candida albicans is a highly prevalent yeast in the oral cavity (11–13) which proliferates and invades host mucosal tissues upon epithelial barrier dysfunction or disruption. C. albicans invades tissues via hypha formation and the production of associated hydrolytic enzymes and virulence factors. While these characteristics may endow *Candida* with a competitive advantage, it is the host's immune competence that ultimately determines whether clearance, colonization, or disease occurs (14).

There has long been a positive association between oral yeast carriage/dysbiosis and epithelial carcinoma (15–19). Notably, higher yeast carriage and diversity are observed in oral cancer patients than in healthy individuals, and oral fungal colonization in OSCC patients is higher on the neoplastic epithelial surface than on adjacent healthy surfaces (20–24). Furthermore, persistent oral candidiasis has been observed to lead to OSCC development in an elderly patient (25). Several other studies have indicated that *Candida* invasion promotes a hyperplastic epithelial response and that untreated *Candida* epithelial lesions may become dysplastic and transform into carcinoma (reviewed in references 25 and 26). Thus, there is strong evidence supporting the idea that *Candida* promotes carcinogenic events in the oral cavity (16, 27–30). However, *Candida* infection in cancer patients may also be considered the consequence of an altered immune status, because both myelosuppression and mucositis enable the development of oral candidiasis (8, 9, 31, 32).

Given the association of oral candidiasis and cancer, in this study we characterized the potential underlying mechanisms for *Candida* enhancing OSCC development and progression. Using *in vitro* and two *in vivo* models, we conclude that C. albicans can facilitate and enhance oncogenic mechanisms during oral cancer.

## RESULTS

### Heat-inactivated *Candida* and zymosan increase *in vitro*-related metastasis.

**FIG 1:**
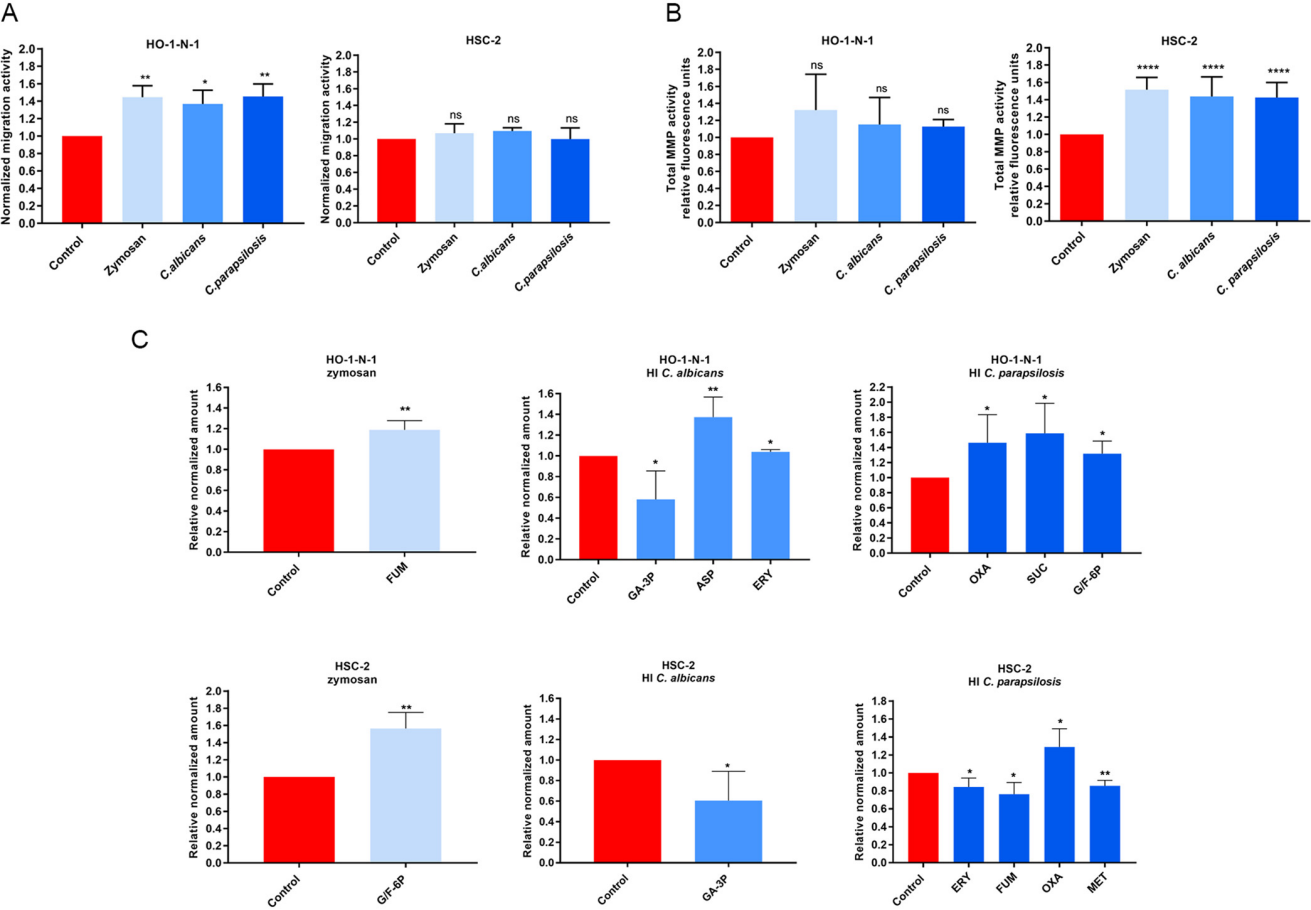
Effects of HI *Candida* and zymosan on HO-1-N-1 and HSC-2 oral squamous cell carcinoma cells *in vitro.* (A) Normalized migration activity of OSCC cells in the presence of HI C. albicans, HI *C parapsilosis*, and zymosan measured by a wound healing assay (*n* = 3). (B) Normalized total secreted matrix metalloproteinase (MMP) activity of OSCC cells in the presence of HI C. albicans, HI C. parapsilosis, and zymosan measured by a total MMP activity kit (*n* = 4). (C) Normalized amounts of metabolites of OSCC cells in the presence of HI C. albicans, HI C. parapsilosis, and zymosan as measured by HPLC-HRMS (*n* = 4). FUM, fumaric acid; GA-3P, glyceraldehyde-3P; ASP, aspartic acid; ERY, erythrose-4P; OXA, oxaloacetic acid; SUC, succinic acid; G/F-6P, glucose/fructose-6p; MET, methionine; control, tumor cells without any treatment. Unpaired *t* test; *, *P* ≤ 0.05; **, *P* ≤ 0.01; ****, *P* ≤ 0.0001. ns, nonsignificant.

To examine whether increased fungal burden affects oral tumor progression, HSC-2 and HO-1-N-1 OSCC cells were treated with zymosan (cell wall component of Saccharomyces cerevisiae), heat-inactivated (HI) Candida albicans, or HI Candida parapsilosis yeast cells. Initially, a wound healing assay at 24 h was used to analyze the invasive capacity of OSCC cells. Cellular movement of HO-1-N-1 cells was significantly enhanced by all three treatments compared to that of the untreated control (zymosan,1.445 ± 0.076; HI C. albicans, 1.369 ± 0.09; HI C. parapsilosis, 1.454 ± 0.083). No significant differences in invasiveness were observed with HSC-2 cells (Fig. 1A). Next, epithelial proliferation during fungal exposure was performed using bromodeoxyuridine (BrdU) proliferation assays, which revealed that HI *Candida* cells do not affect OSCC cell proliferation (see Fig. S1A in the supplemental material).

10.1128/mBio.03144-21.7 DATA SET S1 (A) List of differentially expressed genes (DEGs) in HO-1-N-1 cell line after live Candida albicans treatment. C. albicans-treated cells compared to control. (B) List of DEGs in HO-1-N-1 cell line after live Candida parapsilosis treatment. C. parapsilosis-treated cells compared to control. (C) List of DEGs in HO-1-N-1 cell line after heat-inactivated (HI) Candida albicans treatment. HI C. albicans-treated cells compared to control. (D) List of DEGs in HO-1-N-1 cell line after HI Candida parapsilosis treatment. HI C. parapsilosis-treated cells compared to control. (E) List of DEGs in HO-1-N-1 cell line after zymosan treatment. Zymosan-treated cells compared to control. (F) List of DEGs in HSC-2 cell line after live Candida albicans treatment. C. albicans-treated cells compared to control. (G) List of DEGs in HSC-2 cell line after live Candida parapsilosis treatment. C. parapsilosis-treated cells compared to control. (H) List of DEGs in HSC-2 cell line after HI Candida albicans treatment. HI C. albicans-treated cells compared to control. (I) List of DEGs in HSC-2 cell line after HI Candida parapsilosis treatment. HI C. parapsilosis-treated cells compared to control. (J) List of DEGs in HSC-2 cell line after zymosan treatment. Zymosan-treated cells compared to control. (K) List of DEGs in the *in vivo* tumor samples. OC-OSCC xenograft compared to OSCC xenograft. (L) List of genes (with reference) which are involved in OSCC progression. (M) Primer sequences used in qPCR. (N) The precursor mass, fragment ion mass, polarity, retention time, and fragmentation energy and the lower limit of determination (LLOQ) of the examined metabolites in HPLC-HRMS analysis. Download Data Set S1, XLSX file, 3.6 MB. Copyright © 2022 Vadovics et al. 2022 Vadovics et al. https://creativecommons.org/licenses/by/4.0/ This content is distributed under the terms of the Creative Commons Attribution 4.0 International license.

Remodeling of the extracellular matrix is a critical component of tumor cell adaptability and is attributed to the secretion of several proteases (serine, cysteine, threonine, aspartic acid, and metalloproteinases). In particular, matrix metalloproteinases (MMPs) are key enhancers of tumor dissemination (33). Notably, all fungal treatments significantly elevated secreted MMP activity in HSC-2 cells at 24 h compared to the untreated control (zymosan, 1.516 ± 0.041; HI C. albicans, 1.437 ± 0.06536; HI C. parapsilosis, 1.426 ± 0.057). However, MMP activity was unaltered in HO-1-N-1 cells (Fig. 1B).

Metabolites generated by cancer cells influence the metastatic cascade, affecting the epithelial-mesenchymal transition (EMT), the survival of cancer cells in circulation, and metastatic colonization at distant sites (34). Changes in metabolic activity were examined by analyzing the levels of glycolysis and tricarboxylic acid (TCA) cycle intermediates and certain amino acids (Fig. S1C) by high-performance liquid chromatography coupled with high-resolution mass spectrometry (HPLC-HRMS) after 24 h of treatment. For the HSC-2 cell line, zymosan treatment significantly increased the production of glucose/fructose 6-phosphate (glucose/fructose-6p) (1.564 ± 0.132), while HI C. albicans treatment reduced the concentrations of glyceraldehyde 3-phosphate (GA-3P) (0.605 ± 0.142). HI C. parapsilosis treatment altered levels of erythrose-4P (0.844 ± 0.056), fumaric acid (0.763 ± 0.065), oxaloacetic acid (1.289 ± 0.117), and methionine (0.855 ± 0.031) (Fig. 1C). Nonsignificant changes are shown in Fig. S1C. In HO-1-N-1 cells, zymosan treatment produced a significant change in fumaric acid (1.189 ± 0.044), while HI C. albicans treatment altered the levels of glyceraldehyde-3P (0.580 ± 0.137), aspartic acid (1.374 ± 0.096), and erythrose-4P (1.039 ± 0.015). HI C. parapsilosis treatment altered the production of oxaloacetic acid (1.463 ± 0.214), succinic acid (1.586 ± 0.199), and glucose/fructose-6p (1.317 ± 0.118) (Fig. 1C). Taken together, all three fungal treatments had a significant effect on migration, secreted MMP activity, and oncometabolite production of OSCC cells, which suggests interactions between the tumor cells and fungal components.

10.1128/mBio.03144-21.1 FIG S1 (A) Normalized proliferation activity of OSCC cells in the presence of heat- killed C. albicans, C. parapsilosis, and zymosan measured by BrdU incorporation assay. (B) Normalized proliferation activity of OSCC cells in the presence of live C. albicans and C. parapsilosis measured by BrdU incorporation assay. (C) Normalized amount of metabolites of OSCC cells in the presence of heat-killed C. albicans, C. parapsilosis. and zymosan measured by HPLC-HRMS. (D) Normalized amount of metabolites of OSCC cells in the presence of live C. albicans and C. parapsilosis measured by HPLC-HRMS. Unpaired *t* test; *, *P* ≤ 0.05; **, *P* ≤ 0.01; ***, *P* ≤ 0.001; ****, *P* ≤ 0.0001. Download FIG S1, PDF file, 0.6 MB. Copyright © 2022 Vadovics et al. 2022 Vadovics et al. https://creativecommons.org/licenses/by/4.0/ This content is distributed under the terms of the Creative Commons Attribution 4.0 International license.

### Live *Candida* enhances detachment, MMP activity, and metabolite production of OSCC cells *in vitro*.

**FIG 2:**
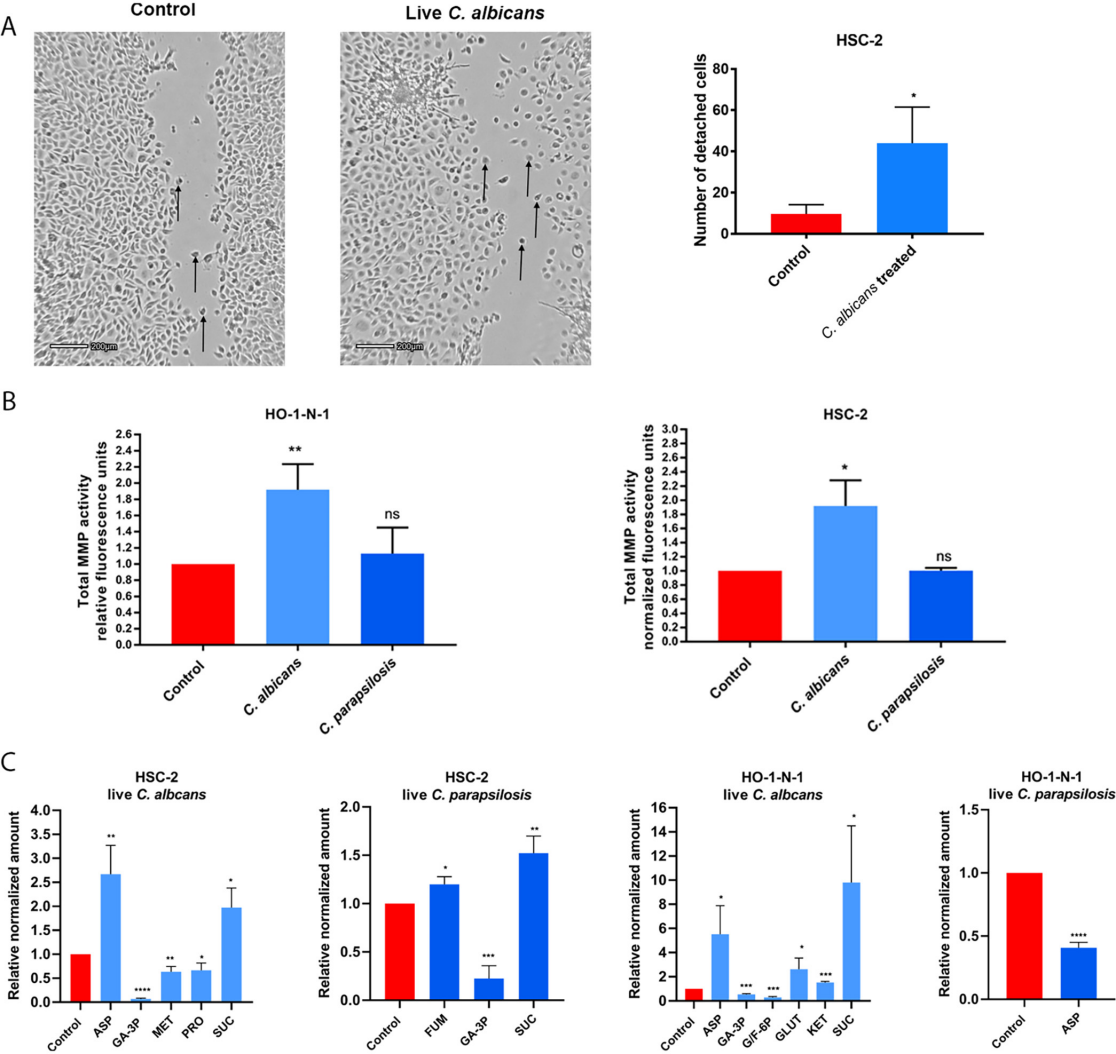
Effects of live *Candida* on HO-1-N-1 and HSC-2 oral squamous cell carcinoma cells *in vitro.* (A) Pictures from time-lapse videos of cellular migration of HSC-2 cells, with arrows pointing to detached cancer cells. The left picture shows the control cells, and the right shows the live C. albicans-treated cells. The graph shows the number of detached cells (*n* = 3). (B) Normalized total secreted matrix metalloproteinase activity of OSCC cells in the presence of live C. albicans and C. parapsilosis as obtained by a total MMP activity kit (*n* = 3). (C) Normalized amounts of metabolites of OSCC cells in the presence of live C. albicans and live C. parapsilosis as measured by HPLC-HRMS (*n* = 3). ASP, aspartic acid; GA-3P, glyceraldehyde-3P; MET, methionine; PRO, proline; SUC, succinic acid; FUM, fumaric acid; G/F-6P, glucose/fructose-6p; GLUT, glutamic acid; KET, α-ketoglutaric acid. Control, tumor cells without any treatment. Unpaired *t* test; *, *P* ≤ 0.05; **, *P* ≤ 0.01; ***, *P* ≤ 0.001; ****, *P* ≤ 0.0001.

After assessing the effects of HI *Candida* infections, we further aimed to examine the potential outcome of live fungal stimuli. While during HI fungal treatment, the triggered host responses are mainly due to direct cell-cell contact, live *Candida* cells could also influence host responses through other “indirect” stimuli, such as their secreted extracellular vesicles, released enzymes, etc. Therefore, we next assessed the effects of live C. albicans and C. parapsilosis on OSCC cultures and recorded the migration of cancer cells on time-lapse video. Live C. albicans, but not live C. parapsilosis, increased the numbers of detached, single HSC-2 cells compared to untreated controls (Fig. 2A; Video S1, S2, and S3). No changes were detected in the HO-1-N-1 cell line (data not shown). Similar to HI *Candida*, live C. albicans and C. parapsilosis did not alter OSCC proliferation as measured by a BrdU assay (Fig. S1B). Secreted total MMP activity was increased in both cancer cell lines with live C. albicans (HSC-2, 1.918 ± 0.209; HO-1-N-1, 1.918 ± 0,183) but not live C. parapsilosis (Fig. 2B).

10.1128/mBio.03144-21.8 VIDEO S1 Control. Migration of HSC-2 cells without any treatment. Download Movie S1, AVI file, 13.7 MB. Copyright © 2022 Vadovics et al. 2022 Vadovics et al. https://creativecommons.org/licenses/by/4.0/ This content is distributed under the terms of the Creative Commons Attribution 4.0 International license.

10.1128/mBio.03144-21.9 VIDEO S2 Migration of HSC-2 cells during C. albicans treatment. Download Movie S2, AVI file, 14.2 MB. Copyright © 2022 Vadovics et al. 2022 Vadovics et al. https://creativecommons.org/licenses/by/4.0/ This content is distributed under the terms of the Creative Commons Attribution 4.0 International license.

10.1128/mBio.03144-21.10 VIDEO S3 Migration of HSC-2 cells during C. parapsilosis treatment. Download Movie S3, AVI file, 9.5 MB. Copyright © 2022 Vadovics et al. 2022 Vadovics et al. https://creativecommons.org/licenses/by/4.0/ This content is distributed under the terms of the Creative Commons Attribution 4.0 International license.

Next, metabolic changes in OSCC cells were measured following treatment with live C. albicans and C. parapsilosis at 24 h. Importantly, no fungal metabolites were detected using our extraction method. In HSC-2 cells, C. albicans significantly altered the secretion of aspartic acid (2.67 ± 0.346), glyceraldehyde-3P (0.068 ± 0.009), methionine (0.634 ± 0.063), proline (0.666 ± 0.087), and succinic acid (1.975 ± 0.234), while C. parapsilosis altered fumaric acid (1.199 ± 0.046), glyceraldehyde-3P (0.225 ± 0.076), and succinic acid (1.523 ± 0.101) secretion. In HO-1-N-1 cells, C. albicans significantly altered the secretion of aspartic acid (5.526 ± 1.667), glyceraldehyde-3P (0.543 ± 0.038), glucose/fructose-6p (0.288 ± 0.047), glutamine (2.616 ± 0.667), α-ketoglutaric acid (1.532 ± 0.051), and succinic acid (9.81 ± 2.709), while C. parapsilosis treatment reduced the level of aspartic acid (0.408 ± 0.024) (Fig. 2C). C. albicans was predominantly in the hyphal form and C. parapsilosis in the yeast form when these assays were performed.

The data demonstrate that live C. albicans induced the most prominent changes in the movement, MMP activity, and metabolite production in OSCC cells compared to live C. parapsilosis or HI *Candida* and zymosan treatments.

### Live C. albicans activates genes and signaling pathways involved in OSCC invasion and metastasis.

To fully dissect the molecular mechanisms induced by fungal stimulation, whole-transcriptome analysis was performed with HSC-2 and HO-1-N-1 cells following exposure to zymosan, HI C. albicans, HI C. parapsilosis, live C. albicans, and live C. parapsilosis. Transcriptome analyses revealed that live C. albicans induced the most significant gene expression changes in OSCC cells (HSC-2, *n* = 2,764; HO-1-N-1, *n* = 137), followed by zymosan (*n* = 19), while live C. parapsilosis and heat-inactivated fungal challenge did not trigger significant gene expression changes in either cell line.

**FIG 3:**
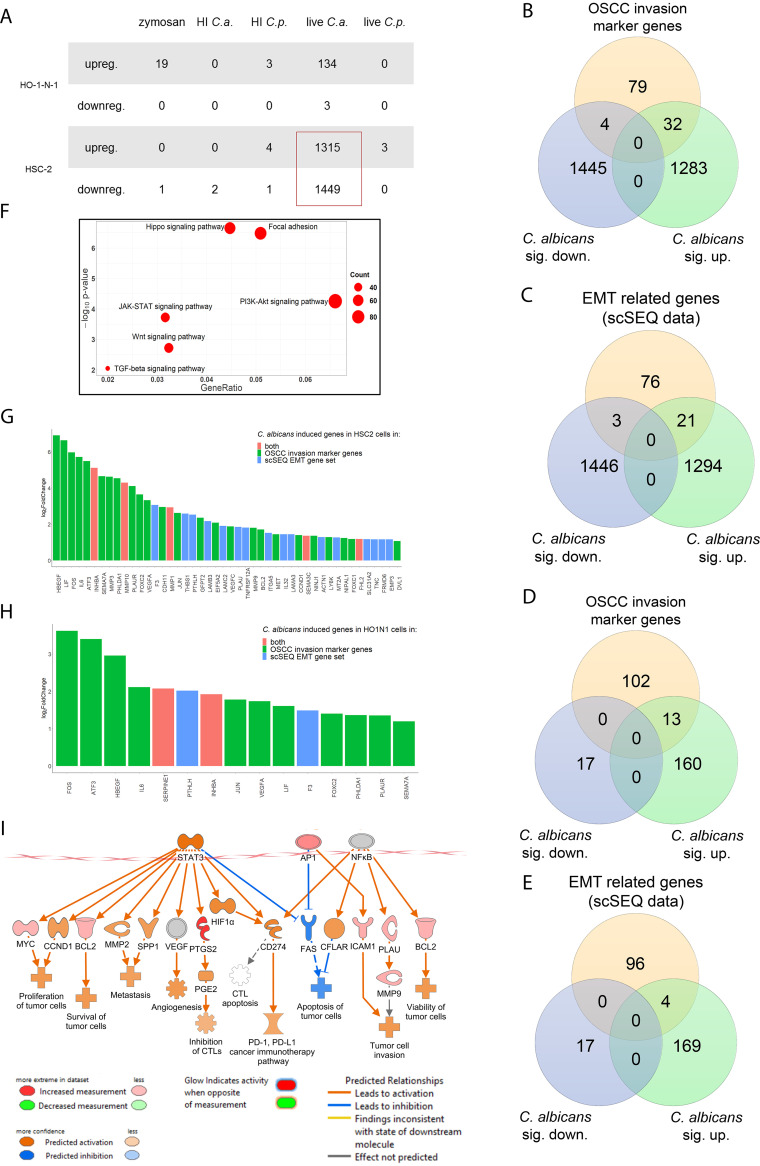
*In vitro* transcriptomic analysis. Candida albicans activates genes and signaling pathways involved in the OSCC metastatic processes. (A) Number of up- or downregulated genes of HSC-2 and HO-1-N-1 cells after different fungal treatments (*n* = 3). C.a., C. albicans; C.p., C. parapsilosis (B) Venn diagram of up- or downregulated genes in HSC-2 cells in the presence of live C. albicans and OSCC invasion marker genes found in the literature. sig., significantly. (C) Venn diagram of up- or downregulated genes in the HSC-2 cell line in the presence of live C. albicans and EMT marker genes in HNSCC according to a single-cell sequencing study. (D) Venn diagram of up- or downregulated genes in HO-1-N-1 cells incubated with live C. albicans and OSCC invasion marker genes found in the literature. (E) Venn diagram of up- or downregulated genes in the HO-1-N-1 cell line in the presence of live C. albicans and EMT marker genes in HNSCC according to a single-cell sequencing study. (F) Signaling pathways that are key regulators of the OSCC invasion processes that were significantly activated in HSC-2 cells in the presence of live C. albicans. (G) Graph showing log_2_ fold change of C. albicans-induced genes in HSC-2 cells involved in OSCC invasion according to the literature and of single-cell sequencing (scSeq) results from 18 patients with HNSCC. Red columns represent OSCC marker genes according to the literature and scSEQ data. (H) Graph showing log_2_ fold change of C. albicans-induced genes in HO-1-N-1 cells involved in OSCC invasion according to the literature and scSEQ data. Red columns represent OSCC marker genes according to the literature and scSEQ data. (I) Causal analyses of the genes for which expression changed in HSC-2 cells after live Candida albicans treatment.

C. albicans evoked a more significant response in HSC-2 (upregulated genes, *n* = 1,315; downregulated genes, *n* = 1,449) than HO-1-N-1 cells (upregulated, *n* = 134; downregulated, *n* = 3) (Fig. 3A; Data Set S1A to J). Notably, in HSC-2 cells, C. albicans triggered significant changes in marker genes previously associated with OSCC invasion (32 upregulated, 4 downregulated) (Data Set S1L) (Fig. 3B). Moreover, another gene set (21 upregulated, 3 downregulated) overlapped with a characteristic profile of EMT derived from single-cell sequencing (scSeq) of 18 patients with head and neck squamous cell carcinoma (HNSCC) (35) (Fig. 3C). Five genes, *INHBA*, *MMP10*, *MMP1*, *SEMA3C*, and *FHL2*, were present in both the OSCC invasion marker gene and scSeq-derived gene data sets (Fig. 3G). Furthermore, even though the HO-1-N-1 response was modest compared to HSC-2, 13 OSCC invasion marker genes (Fig. 3D) and four EMT genes (Fig. 3E) were also upregulated after live C. albicans stimulus. Two of these genes, *SERPINE1* and *INHBA*, overlapped with the OSCC invasion marker genes and EMT subsets (Fig. 3H).

KEGG pathway analysis of HSC-2 data sets revealed significant activation of several pathways associated with OSCC metastasis development, including Hippo signaling, focal adhesion, JAK-STAT, PI3K-Akt, Wnt, and TGFβ pathways (Fig. 3F) (36–40). Ingenuity pathway analysis (IPA) with built-in causal analyses was also used to investigate activation patterns of several intracellular signaling pathways based on the coherent regulation of their molecular elements. IPAs predicted the activation of tumor-related pathways, including the tumor microenvironment pathway, as well as the significant activation of several prognostic features, such as metastasis, invasion, angiogenesis, and proliferation of tumors based on the C. albicans stimulus-derived differentially expressed genes (DEGs) (Fig. 3I). Interestingly, *DLST* and *SUCLA* gene expression was increased, and these genes are involved in succinic acid metabolism. Furthermore, *ASNSD1* and *GOT1* genes were also upregulated and are involved in aspartic acid synthetic processes (Fig. S4). Transcriptomic gene expression data was validated by quantitative PCR (qPCR) (Fig. S2 and S4). Together, these data support the notion that live C. albicans, but not live C. parapsilosis, HI *Candida*, or zymosan, enhances the metastatic features of OSCC cells.

10.1128/mBio.03144-21.2 FIG S2 Validation of C. albicans activated signaling pathways. Validation was performed by qPCR analysis of pathway components. (A) PI3K-Akt signaling pathway; (B) TGF-β/SMAD; (C) Hippo signaling pathway; (D) Wnt signaling pathway; (E) focal adhesion pathway. Unpaired *t* test; *, *P* ≤ 0.05; **, *P* ≤ 0.01. Download FIG S2, PDF file, 0.4 MB. Copyright © 2022 Vadovics et al. 2022 Vadovics et al. https://creativecommons.org/licenses/by/4.0/ This content is distributed under the terms of the Creative Commons Attribution 4.0 International license.

10.1128/mBio.03144-21.3 FIG S3 (A) Comparison of *Candida-*induced genes in HSC-2 cell line to genes involved in different tumor progression processes. Differentially expressed gene list derived from an OSCC single-cell sequencing study. (B) Comparison of *Candida-*induced genes in HO-1-N-1 cell line to genes involved in different tumor progression processes. Differentially expressed gene list derived from an OSCC single-cell sequencing study. (C) Live C. albicans-induced genes in both (HSC-2 and HO-1-N-1) cell lines, which are involved in OSCC progression. OSCC progression marker gene list derived from literature and OSCC single-cell sequencing study. Download FIG S3, PDF file, 0.5 MB. Copyright © 2022 Vadovics et al. 2022 Vadovics et al. https://creativecommons.org/licenses/by/4.0/ This content is distributed under the terms of the Creative Commons Attribution 4.0 International license.

10.1128/mBio.03144-21.4 FIG S4 (A) Validation of transcriptomic data by qPCR. Unpaired *t* test; *, *P* ≤ 0.05; **, *P* ≤ 0.01; ***, *P* ≤ 0.001; ****, *P* ≤ 0.0001. Download FIG S4, PDF file, 0.3 MB. Copyright © 2022 Vadovics et al. 2022 Vadovics et al. https://creativecommons.org/licenses/by/4.0/ This content is distributed under the terms of the Creative Commons Attribution 4.0 International license.

### Establishment of a novel *in vivo* mouse xenograft model of OSCC and oral candidiasis.

**FIG 4:**
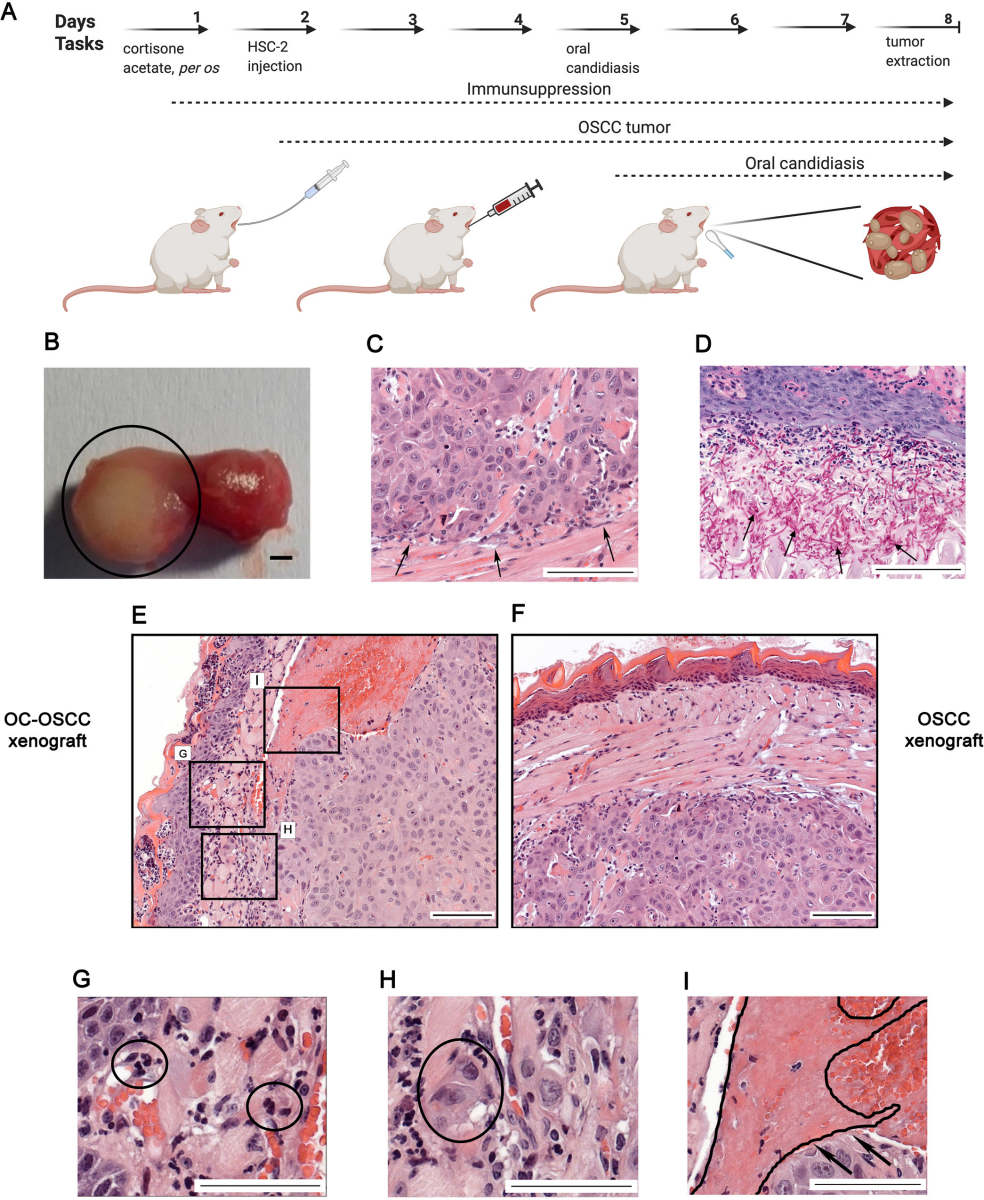
A new *in vivo* mouse model for the investigation of oropharyngeal candidiasis on the progression of OSCC. (A) Schematic figure of mouse xenograft for the investigation of the effect of C. albicans on the progression of OSCC. Immunosuppression and injection of human HSC-2 OSCC cells into the tongue of mice (OSCC xenograft). OSCC xenograft and oral candidiasis (OC-OSCC xenograft). The cartoon was produced by BioRender. (B) Representative mouse tongue on the 8th day of the experiment (7 days after tumor cell injection). The circle highlights the tumor. Scale bar, 1 mm. (C) Histopathological image of the tumor on the 8th day, with black arrows indicating the tumor edge. Scale bar, 100 μm. (D) Histopathological examination of the tumor on the 8th day (7 days after tumor injection and 3 days postinfection), with black arrows indicating the fungal hyphae in the mucosa. Scale bar, 100 μm. (E) Histopathological picture of the tumor on the 8th day after HSC-2 injection and oral candidiasis. Scale bar, 100 μm. (F) Histopathological picture of the tongue on the 8th day after HSC-2 injection. Scale bar, 100 μm. (G) Infiltrating immune cells in OC-OSCC xenograft samples indicating that C. albicans caused inflammation. Scale bar, 100 μm. (H) Detached budding tumor cells in OC-OSCC xenograft samples indicating epithelial-to-mesenchymal transition. Scale bar, 100 μm. (I) Thrombosis in OC-OSCC xenograft samples. Scale bar, 100 μm. Created by BioRender.

In order to validate our *in vitro* results, we developed a novel *in vivo* mouse model of OSCC and oral candidiasis. As C. albicans cells are not in direct contact with HSC-2 cells in this model, the indirect effect of oral candidiasis on OSCC progression can be examined. To mimic the immunological condition of patients caused by chemoradiotherapy, cortisone acetate was administered to 6- to 8-week-old BALB/c mice to induce an immunosuppressed condition for subsequent tumor cell injection. As HSC-2 cells were more responsive to fungal presence than HO-1-N-1 cells, 1 × 10^6^ HSC-2 cells were injected into mouse tongues to initiate OSCC development. A fully developed tumor formed by day 3 after HSC-2 injection. After confirmation of tumor presence, calcium alginate swabs saturated in 1 × 10^9^/mL C. albicans suspension for 5 min were placed under the tongue of each mouse for 75 min on day 5 (3 days after tumor cell injection). Mice were terminated on day 8 based upon clinical observation scores and weight loss (25%) (Fig. S5C). On day 8, the average tumor size was 5 mm in diameter (Fig. 4B). Hematoxylin-eosin (H&E) staining was performed for histopathological analyses (Fig. 4C). Fungal hyphae were detected in the mucosa following periodic acid-Schiff (PAS) staining (Fig. 4D). The oral C. albicans burden was 10^5^ to 10^6^ CFU per g tissue, which is comparable with that reported in the literature of oral candidiasis models (41).

10.1128/mBio.03144-21.5 FIG S5 (A) Histopathological samples of *Candida*-colonized and *Candida*-free tumors, analyzed and scored manually by a pathologist after H&E staining. (B) Causal analyses of the genes whose expression changed in OC-OSCC samples after oral candidiasis. (C) Weight loss of the animals after HSC-2 tumor cell injection (OSCC xenograft) and HSC-2 injection combined with oral candidiasis (OC-OSCC xenograft). (D) p63 staining of the histopathological samples (animals 2, 3, and 4). (E) E-cadherin staining of the histopathological samples (animals 2, 3, and 4). (F) Vimentin staining of the histopathological samples (animals 2, 3, and 4). Download FIG S5, PDF file, 0.5 MB. Copyright © 2022 Vadovics et al. 2022 Vadovics et al. https://creativecommons.org/licenses/by/4.0/ This content is distributed under the terms of the Creative Commons Attribution 4.0 International license.

### Oral candidiasis enhances the progression of OSCC *in vivo*.

To investigate the effect of increased yeast burden on OSCC progression, two animal groups were compared: a control group received cortisone acetate and HSC-2 tumor cells (OSCC xenograft), while the other group received cortisone acetate, HSC-2 cells, and C. albicans for the development of oral candidiasis (OC-OSCC xenograft). Each group (OSCC xenograft and OC-OSCC xenograft) comprised 16 animals. Four mice were applied for transcriptome analysis and 4 for CFU analysis from each group (OSCC xenograft and OC-OSCC xenograft). For histopathological analysis, 8 mice were applied from each group. CFU analysis was applied for the validation of successful oral candidiasis establishment in the OC-OSCC xenograft group. Histopathological samples from both groups were analyzed and scored manually in a blinded manner by a pathologist after H&E staining for the identification of inflammation, necrosis, infiltrating or pushing tumor edge, EMT, invasion markers, and signs and symptoms of thrombosis and peritumoral inflammation (Fig. S5A). The EMT and budding score number of tumor cells were higher in 5/8 samples in OC-OSCC xenograft tumors than in OSCC xenograft samples, where no high EMT/budding scores were detected. Thrombosis was also detected in 5/8 OC-OSCC xenograft tumors. Only 1 sample showed thrombosis in OSCC xenograft controls.

Infiltrating immune cells were detected within the OC-OSCC xenograft samples, indicating C. albicans-induced inflammatory responses (Fig. 4G). Images indicative of EMT were identified, as represented by the increased number of detached individual tumor cells (Fig. 4H). Ki-67 staining was performed to analyze the proliferation activity of the tumor cells *in vivo*. No difference could be detected between the C. albicans-infected (OC-OSCC xenograft) and OSCC xenograft group (data not shown). Taken together, histopathological scoring suggests that oral candidiasis may drive OSCC progression events.

### Oral candidiasis increases p63 and vimentin expression and decreases E-cadherin expression in OSCC histopathological specimens.

**FIG 5:**
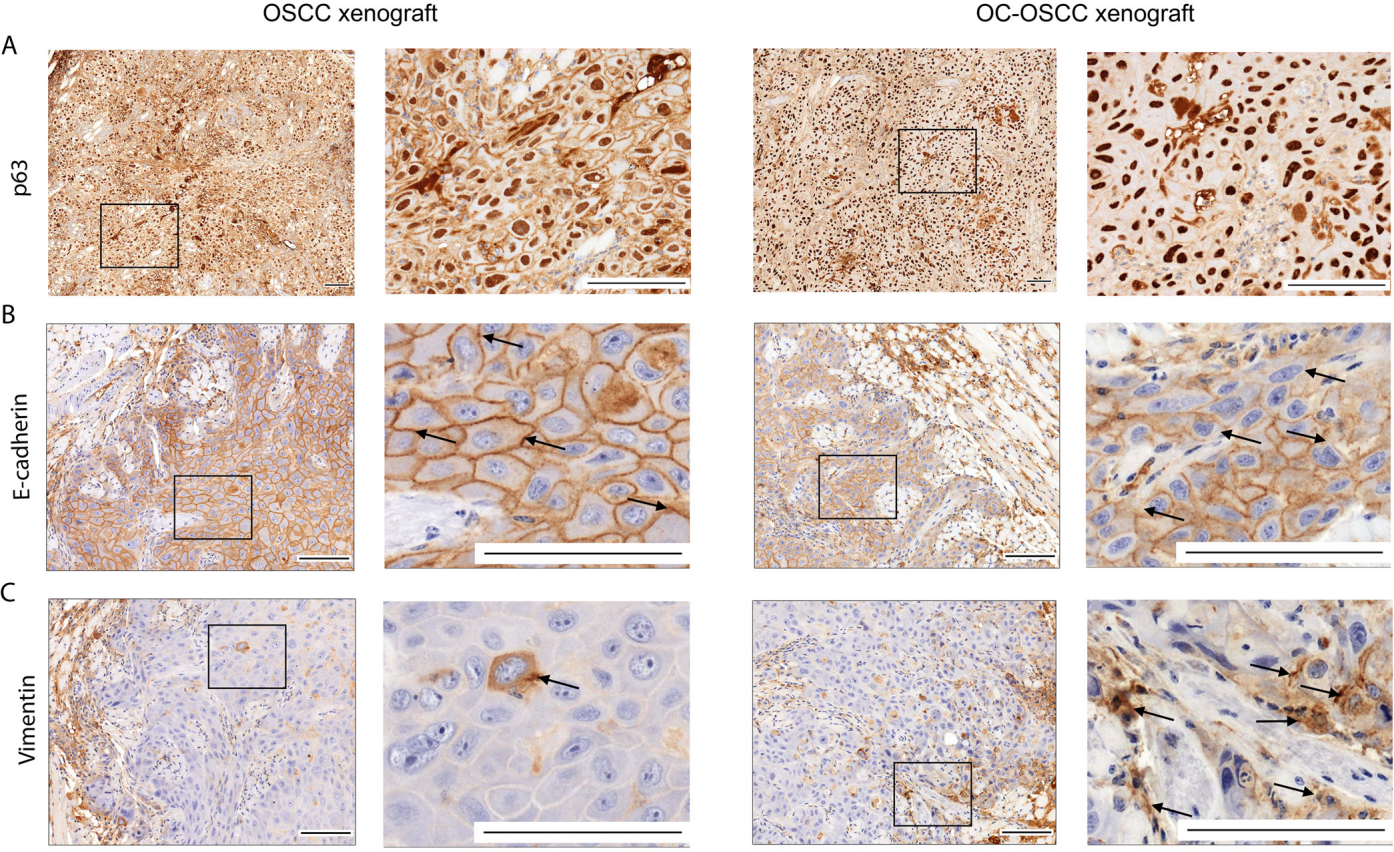
Histopathological staining of OSCC and OC-OSCC xenograft tumor samples: p63 staining (A), E-cadherin staining (B), and vimentin staining (C). Scale bars, 100 μm. *n* = 8/group. Squares indicate the magnified sections (right panels per mice model) of each tissue sample. Arrows indicate the E-cadherin positive (upper panels) and vimentin positive (lower panels) cells.

p63 expression is a reliable indicator in histological grading and is an early marker of a poor OSCC prognosis. Analysis of OC-OSCC xenograft samples showed that C. albicans increased p63 expression and localization to the nucleus compared to uninfected tissues (Fig. 5A). Next, we analyzed for the expression of EMT markers E-cadherin and vimentin. Reduced E-cadherin expression was observed in cell membranes of OC-OSCC xenograft samples compared with OSCC xenograft tumors (Fig. 5B). Correspondingly, vimentin-positive cells were notably increased in OC-OSCC xenograft sections compared with OSCC xenograft sections (Fig. 5C; Fig. S5D, E, and F). The data indicate that C. albicans can drive an EMT phenotype.

### Oral candidiasis enhances the expression of genes involved in OSCC progression *in vivo*.

**FIG 6:**
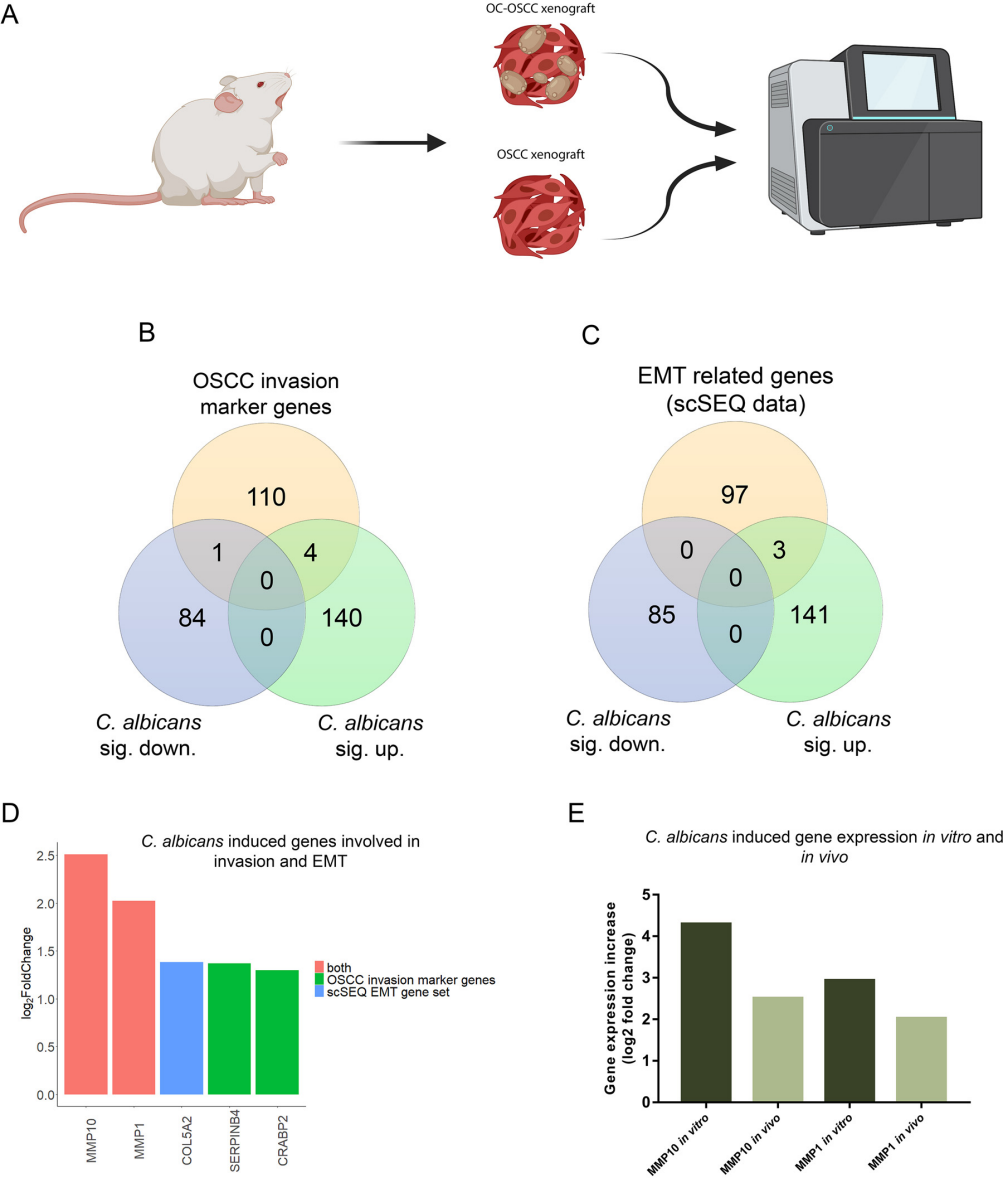
Transcriptomic analysis of *in vivo* tumor samples followed by oral candidiasis. (A) Schematic figure of mRNA sequencing of OSCC xenograft and OC-OSCC xenograft tumor samples. The cartoon was produced by BioRender. (B) Venn diagram of up- or downregulated genes in HSC-2 cells in the presence of live C. albicans and OSCC invasion marker genes described in the literature. (C) Venn diagram of up- or downregulated genes in HSC-2 cell line in the presence of live C. albicans and EMT marker genes in HNSCC according to a single-cell sequencing study. (D) Graph showing log_2_ fold change of C. albicans-induced genes in HSC-2 cells involved in OSCC invasion according to the literature and single-cell sequencing data. Red columns represent OSCC marker genes according to both the literature and single-cell sequencing (scSeq) results from 18 patients with HNSCC. (E) Tumor invasion genes showing upregulated expression both *in vitro* and *in vivo*. For transcriptomic analysis, *n* = 4. Created by BioRender.

To analyze the molecular mechanisms behind the histopathological results, transcriptomic analysis of OC-OSCC and OSCC xenograft tumor samples was performed (Fig. 6A). OC-OSCC xenografts displayed expression changes in 229 genes (144 upregulated and 85 downregulated) (Data Set S1K). Among these, 5 genes (*MMP10*, *MMP1*, *SERPINB4*, and *CRABP2* upregulated; *MMP7* downregulated) are predicted to be involved in OSCC invasion (Fig. 6B), while 3 genes (*MMP10*, *MMP1*, and *COL5A2* upregulated) are associated with EMT regulation (Fig. 6C). Notably, *MMP10* and *MMP1* were present in both subsets (Fig. 6D) and were also upregulated in our *in vitro* and *in vivo* models (Fig. 6E). *In vitro MMP1* and *MMP10* expression increase was validated by Western blot analysis (Fig. S6C, D, E, and F). The data indicate that C. albicans induces a gene subset predicted to be involved in OSCC invasion.

10.1128/mBio.03144-21.6 FIG S6 (A) qPCR curve of TAp63 and ΔNp63+TAp63 splice variants. The first primer pair was designed close to the C-terminal region, and the second primer pair in the N-terminal region. The first primer pair amplifies both splice variant groups (ΔNp63+TAp63), and the second primer pair amplifies only the splice variant possessing the N-terminal region (TAp63). (B) Quantification cycle (qC) value of the transcript variants. (C) Western blot analysis of MMP10 protein (MOI, tumor cells to fungal cells). (D) Western blot analysis of MMP1 protein (MOI, tumor cells to fungal cells). (E) Densitometry data generated from MMP10 Western blot results. (F) Densitometry data generated from MMP1 Western blot results. Download FIG S6, PDF file, 0.3 MB. Copyright © 2022 Vadovics et al. 2022 Vadovics et al. https://creativecommons.org/licenses/by/4.0/ This content is distributed under the terms of the Creative Commons Attribution 4.0 International license.

### C. albicans affects genes related to carcinogenesis in OKF6/TERT2 nonmalignant epithelial cells *in vitro*.

**FIG 7:**
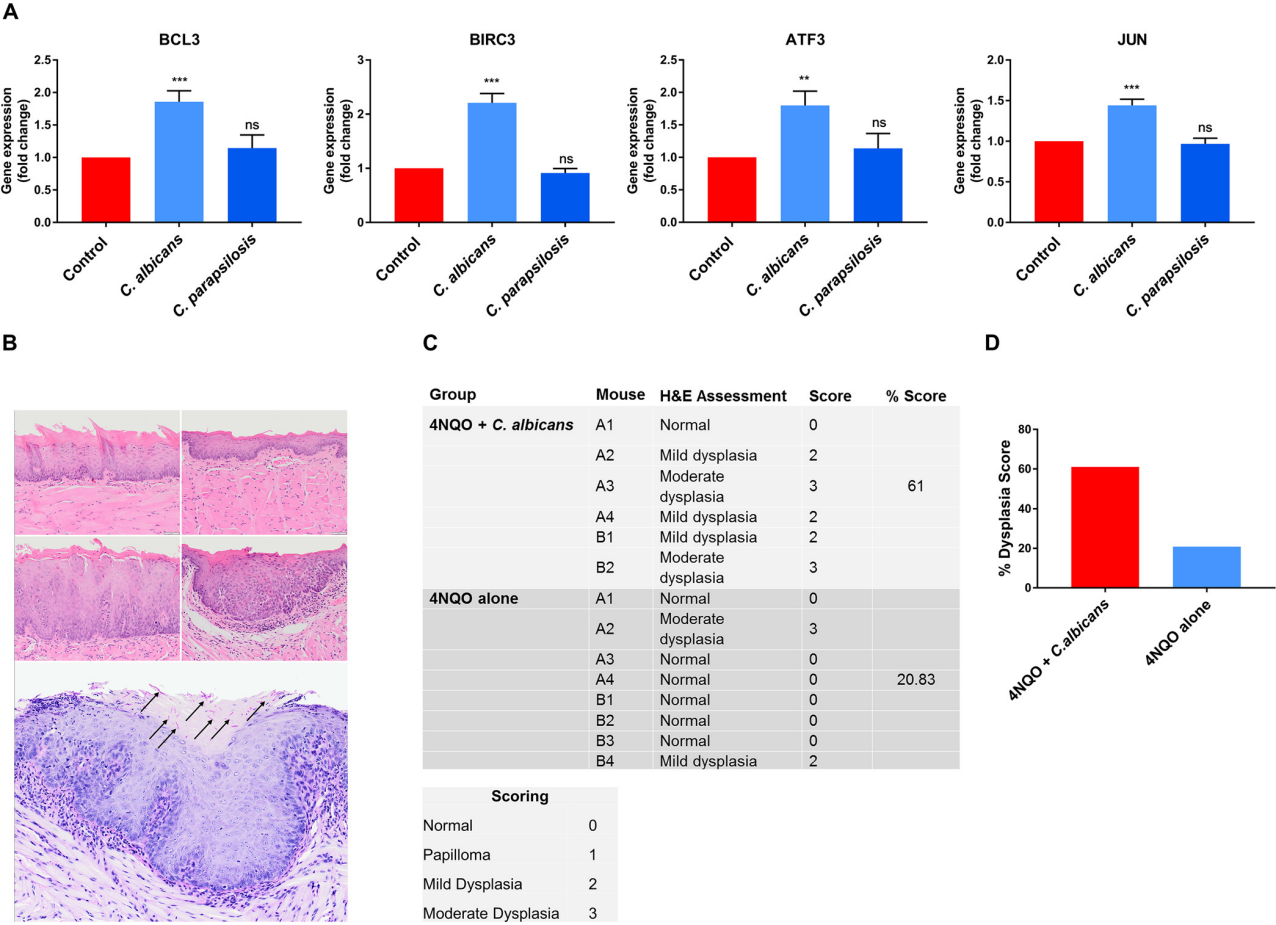
(A) Gene expression of carcinogens in OKF6/TERT2 immortalized cells; (B to D) C. albicans-infected mice exhibited enhanced dysplastic tongue features in the 4NQO model. (A) qPCR results of selected carcinogens in OKF6/TERT2 immortalized cells. (B) Representative photomicrographs of murine oral mucosa demonstrating normal dorsal tongue (top row, left), normal ventral tongue (top row, right), mild epithelial dysplasia on dorsal tongue (middle row, left), and moderate epithelial dysplasia on ventral tongue (middle row, right) (H&E; scale bar, 50 μm). C. albicans hyphae (black arrows) were present in the keratin layer overlying a focus of moderate dysplasia (bottom panel). (C) Each mouse tongue was assessed and graded for atypical architectural and cytological criteria by a qualified histopathologist (normal = 0, papilloma = 1, mild dysplasia = 2, moderate dysplasia = 3), and the percentage score (sum of each group divided by the highest possible score) was calculated per group. (D) Graphical representation of panel C showing the dysplasia score. *n* = 6 for the 4NQO plus C. albicans group; *n* = 8 for the 4NQO-alone group. Scale bar, 50 μm. Unpaired *t* test; **, *P* ≤ 0.01; ***, *P* ≤ 0.001.

The above data show that C. albicans upregulates oncogenes *in vitro* and drives a more malignant phenotype in cancer cells *in vitro* and *in vivo.* We thus wanted to determine whether C. albicans also had carcinogenic potential in normal epithelium. Therefore, we further investigated oral epithelial responses to C. albicans and C. parapsilosis using the oral epithelial cell line OKF6/TERT2, which is a telomerase-deficient line derived from a healthy individual. We recently utilized OKF6/TERT2 cells to investigate non-tumor host cell responses and their microRNA (miRNA) regulatory processes (in epithelial cells) in the presence of C. albicans and C. parapsilosis. Based on these previously acquired sequencing data (42), we selected 4 oncogenes (*BCL3*, *BIRC3*, *ATF3* and *JUN*) (37, 43, 44) potentially regulated by C. albicans and C. parapsilosis. The expression of these genes was analyzed in OKF6/TERT2 cells at 12 h by qPCR after C. albicans and C. parapsilosis stimulus. Only C. albicans significantly upregulated *BLC3*, *BIRC3*, *ATF3*, and *JUN* oncogenes, indicating that C. albicans also contributes to oncogenetic processes in nonmalignant oral epithelial cells (Fig. 7A).

### C. albicans drives oncogenic properties *in vivo* based on the 4NQO mouse model.

Our *in vivo* xenograft OSCC model data and *in vitro* transcriptomic and qPCR data strongly suggest that C. albicans may drive an oncogenic phenotype. However, the limitations of the xenograft OSCC model are its short time span (3 days) and that it addresses only tumor aggressiveness in already established cancer cells. While C. albicans may promote oncogenesis, in humans this is unlikely to occur during short-term colonization episodes, but chronic exposure to fungal virulence factors and metabolites during long-term infections may synergize with other risk factors to promote oncogenesis. To investigate this, we utilized the long-term 4-nitroquinoline 1-oxide (4NQO) mouse model to assess whether topical infection/colonization with C. albicans may directly potentiate an oncogenic phenotype (45). 4NQO is a carcinogen with a mode of activity similar to that of tobacco by-products but when used at low doses establishes conditions that allow us to investigate whether C. albicans can promote stepwise oncogenesis (from normal, through increasing severity of dysplasia, to invasive carcinoma).

BALB/c mice were given a low dose of 4NQO in the drinking water for 8 weeks, followed by 1 week of 0.1% tetracycline (TCN) to condition the epithelium. Mice were then infected with C. albicans (6 × 10^8^ yeast cells/mL) three times at week 10 to establish infection. Tetracycline (0.01%) and C. albicans (6 × 10^4^ yeast cells/mL) were administered via drinking water for a further 10 weeks. At week 12 (2 weeks following C. albicans infection), 2 mice per group were euthanized and their tongues were assessed. Both C. albicans-infected mice showed mild dysplasia (Fig. 7B, middle panels) compared to the uninfected group (Fig. 7B, top panels). At week 20, the remaining mice were euthanized. Mouse tongues were assessed and graded for atypical architectural and cytological criteria by a qualified histopathologist (normal = 0, papilloma = 1, mild dysplasia = 2, moderate dysplasia = 3), and a percentage score was calculated for each experimental group. Notably, 5/6 C. albicans-infected mouse tongues showed either mild or moderate dysplasia with a histological clinical score of 61%, with only 2/8 uninfected mice showing mild or moderate dysplasia with a histological clinical score of ∼21% (Fig. 7C and D). When C. albicans-infected mouse tongues containing dysplasia were subjected to PAS-stained step sectioning, fungal hyphae were identified in 1/5 mouse tongues. Interestingly, in this case, C. albicans colocalized with a focus of moderate dysplasia (Fig. 7B). These findings support the notion that persistent infection with C. albicans can promote stepwise oncogenesis in healthy oral epithelial cells in the presence of predisposing environmental conditions.

## DISCUSSION

OSCC is associated with the presence of oral candidiasis, but whether this is a cause rather than a causative relationship is unclear (15, 17–19, 46–48). We previously showed that the diversity of the oral fungal microflora of OSCC patients is remarkably different from that of healthy individuals, with significantly increased fungal burden and diversity in patients with oral tumors. Furthermore, oral fungal colonization in patients with OSCC is higher on neoplastic epithelial surfaces than on healthy surfaces, which indicates a positive association between oral yeast carriage and epithelial carcinoma (20). *Candida* might induce carcinogenesis by the production of carcinogenic compounds such as nitrosamines (49). These carcinogens bind to bases, phosphate residues, and/or hydrogen bonding sites of DNA that could interfere with DNA replication. Induced point mutations might activate oncogenes and contribute to the development of oral cancer. Accumulation of acetaldehyde, a by-product of ethanol metabolism, is also considered to be carcinogenic, and the induction of proinflammatory cytokines could further contribute to oral cancer development (50), both of which are triggered by the presence of *Candida* cells.

C. albicans and C. parapsilosis both are common commensals of the oral cavity (51). Both are also opportunistic human-pathogenic fungi, although C. albicans is more frequently associated with oral candidiasis than C. parapsilosis (52). C. albicans is polymorphic, due to its ability to form hyphae and/or pseudohyphae (53). C. parapsilosis does not produce true hyphae but can generate pseudohyphae that are characteristically large and curved (54). Hypha formation is critical for host cell damage and immune activation, which are both driven by the secretion of candidalysin, a peptide toxin. Candidalysin damages epithelial membranes and activates several signaling cascades, including the epidermal growth factor receptor (EGFR) pathway (55) which is strongly associated with oral/epithelial cancers (56). Thus, in this study, we aimed to examine whether *Candida* pathogens and their components could potentiate OSCC progression.

*In vitro* experiments using two OSCC cell lines indicated that heat-killed fungi and zymosan are able to induce a moderate amount of cell migration, secreted MMP activity, and oncometabolite production of OSCC cells. However, live C. albicans, but not live C. parapsilosis, was a major inducer of these oncogenic phenotypes. Notably, matrix metalloproteinases (MMPs) are essential for tumor invasion and metastasis, as their secretion degrades components of the extracellular matrix elements, facilitating the migration of individual malignantly transformed cells (57). The transcriptomic data also detected a significant increase in MMP1, MMP10, MMP3, and MMP9 expression. This suggests that C. albicans hypha formation, induction of cellular damage, and activation of epithelial inflammatory and metabolic responses may be critical drivers of MMP activity, tumor invasion, and oncogenic progression. This is also supported by previous findings indicating that the hypha-specific toxin candidalysin induces MMP activity, leading to EGFR activation in the TR146 buccal carcinoma cell line (55).

Metastatic tumor cells have unique metabolic profiles (58). For this reason, we examined the concentrations of glycolysis, TCA cycle intermediates, and some amino acids using HPLC-HRMS with and without exposure to fungal stimuli. Only live C. albicans had a significant effect on metabolic profiles, leading to increased amounts of aspartic acid and succinic acid and decreased the amount of glyceraldehyde-3P (GA-3P) in both OSCC cell lines. Transcriptomic and qPCR data supported these findings. Notably, gene expression of *GOT1*, *DLST*, and *SUCLA2*, involved in aspartic and succinic acid synthesis, was significantly increased. Succinate may drive tumorigenesis through multiple mechanisms, and accumulated succinate can inhibit prolyl hydroxylase, which is responsible for hydroxylation of HIF1α, causing its degradation. Therefore, succinate accumulation through inhibition of prolyl hydroxylase causes HIF1α stabilization and its translocation to the nucleus, which might enhance angiogenesis, resistance against apoptosis, and the activation of genes involved in tumor invasion (59). Furthermore, secreted tumor-derived succinate belongs to a novel class of cancer progression factors, inhibiting tumor-associated macrophage polarization and promoting tumorigenic signaling through PI3K/AKT and HIF1α (60). In addition, aspartate is a limiting metabolite for cancer cell proliferation and tumor growth under hypoxia (61), suggesting that C. albicans-induced aspartic acid increase may facilitate OSCC progression. We assume that the reduced GA-3P levels is the result of increased glyceraldehyde-3-phosphate dehydrogenase (GAPDH) activity. GAPDH catalyzes the redox reaction in the glycolytic pathway by converting GA-3P to 1,3-bisphosphoglycerate with a reduction of NAD^+^ to NADH. GAPDH promotes cancer growth and metastasis through upregulation of SNAIL expression (62). Therefore, the increase in succinic and aspartic acids, together with the decrease in GA-3P, suggests that C. albicans enhances OSCC progression by altering complex metabolic processes in tumor cells.

Transcriptome analysis supported the *in vitro* findings, with live C. albicans significantly altering invasive features of OSCC cells. HSC-2 cells exhibited more prominent responses to C. albicans than HO-1-N-1 cells. However, in both cell lines, we observed a significant increase in expression of 14 genes involved in OSCC invasion and metastasis regulation (*ATF3*, *F3*, *FOS*, *FOXC2*, *HBEGF*, *IL-6*, *INHBA*, *JUN*, *LIF*, *PHLDA1*, *PLAUR*, *PTHLH*, *SEMA7A*, and *VEGFA*). This conforms with and extends previous DNA microarray data describing the impact of C. albicans on reconstituted oral epithelium generated using the TR146 cell line (63), where similar signaling pathways (NF-κB, MAPK, PI3K/Akt) and genes (*ATF3*, *FOS*, *FOXC2*, *HBEGF*, *IL-6*, *JUN*, *LIF*, *PHLDA1*) were significantly increased. All together, we found 1,020 genes and 19 cancer-promoting genes with an altered expression in the presence of C. albicans in both TR146 and HSC-2 cells. The data indicate that C. albicans induces a subset of genes and signaling pathways predicted to be involved in OSCC invasion and progression.

To confirm whether C. albicans enhanced the invasive and potential metastatic activity of HSC-2 OSCC cells *in vivo*, we developed a novel mouse model of OSCC and oral candidiasis. While metastatic events could not be determined given the short time frame of this model (3 days), invasion and the initiation of metastatic events on the molecular level are detectable, namely, in gene expression change and detection of EMT markers. In the OC-OSCC tongues, infiltrating immune cells with concomitant severe inflammation caused by C. albicans was observed on the mucosa. Signs of inflammation could also be observed in the tumor tissue. Inflammatory cells promote the development, advancement, and metastasis of cancer by producing tumor-promoting cytokines. Furthermore, inflammation can alter the tumor microenvironment by inducing growth, survival, extracellular matrix, proangiogenic factors, and reactive oxygen species (64). Indeed, C. albicans and notably candidalysin can induce fibroblast growth factor 2 release and angiogenesis (65).

EMT plays a vital role in invasion and metastasis of cancer cells (66). Thrombosis was also detected in five OC-OSCC xenograft samples. The positive correlation between thrombosis and tumor invasion is well established, but the precise pathological processes are unclear (67). To evaluate whether C. albicans enhances metastatic events, we performed p63 staining on histopathological samples. p63 is the protein encoded by the *TP63* gene, a *TP53* gene homolog known for its role in cell cycle regulation and tumor differentiation. Its overexpression is associated with a poor prognosis of head and neck squamous cell carcinoma (68). p63 has two different promoter domains that produce TAp63, which includes an NH_2_-terminal transactivation domain, and ΔNp63, which lacks an NH_2_-terminal domain. ΔNp63 enhances EMT events during tumor progression by competing with TAp63 and p53 for binding sites (68, 69). In OC-OSCC xenografts, p63 expression and localization to the nucleus were higher than in the OSCC xenografts. We validated this by qPCR in HSC-2 cells in which the ΔNp63 (lacks N-terminal domain) transcript variant was found (Fig. S6A and B). EMT progression in OC-OSCC xenografts was confirmed with vimentin and E-cadherin staining. Vimentin is a cytoskeletal protein that is expressed in mesenchymal cells (fibroblasts, endothelial cells, lymphocytes) but not in healthy epithelial cells, and its upregulation in tumors is linked with lymph node metastasis (70). Increased vimentin expression is associated with a poor prognosis in OSCC (71–73). In OC-OSCC histopathological sections, we detected more vimentin-positive cells than in OSCC samples. E-cadherin, a 120-kDa transmembrane receptor involved in cell-cell adhesion, plays an important role in cell polarity and is involved in several signal transduction pathways (e.g., induction of apoptosis, growth factor receptor activation) (74, 75). Reduced expression of E-cadherin is a reliable indicator of increased invasiveness of OSCCs (76). OC-OSCC tumor samples showed reduced E-cadherin membrane positivity in comparison to OSCC tumor samples. The increased p63 and vimentin expression and decreased E-cadherin expression in the *Candida*-colonized tumors indicate that C. albicans can drive an EMT phenotype, which may lead to a poor prognosis for oral candidiasis-associated OSCC.

Importantly, transcriptome analysis of the *in vivo* OC-OSCC samples revealed that C. albicans induced effects under these *in vivo* conditions similar to those in the *in vitro* setting. Also, the expression of MMP1, MMP10, COL5A2, SERPINB4, and CRABP2 was increased under both conditions, confirming that oral candidiasis may drive OSCC progression events *in vivo*.

The mouse model of OSCC and oral candidiasis indicated that C. albicans could promote invasion and the initiation of metastatic events on the molecular level. However, the disadvantage of this model is that oncogenic progression-related mechanisms induced by C. albicans could only be assessed over 3 days. Therefore, we additionally utilized the long-term 4NQO murine model (20 weeks) that enabled a more robust analysis of whether C. albicans can drive oncogenic mechanisms. Correspondingly, we demonstrated that C. albicans-infected mouse tongues showed either mild or moderate dysplasia with a histological clinical score of 60%, with uninfected mice showing a histological clinical score of ∼21%. A previous study also showed that mice exposed to both 4NQO and C. albicans developed oral dysplastic lesions in this model, together with increased expression of Ki-67 and p16, two cell cycle-associated proteins frequently deregulated in oral dysplasia (45). Thus, the combined findings of the two *in vivo* models support the notion that persistent infection with C. albicans promotes stepwise oncogenesis in healthy oral epithelial cells in the presence of predisposing environmental conditions.

The upregulation of oncogenes in OKF6/TERT2 cells and the increased dysplastic change of normal oral epithelium in 4NQO-treated mice by C. albicans demonstrated in our study have clinical implications. High fungal burdens and/or identification of hyphal forms in dysplastic lesions may indicate a greater risk of malignant transformation. Therefore, when present, effective elimination of C. albicans should form part of the preventative treatment regime in patients at risk of developing OSCC. We also show that C. albicans induces a more aggressive malignant phenotype in OSCC cells *in vitro* and *in vivo*, raising the possibility that the fungus may adversely affect the prognosis of patients with established tumors. Patients with OSCC are at particular risk of developing second primary head and neck tumors (77). Since these patients are also more susceptible to C. albicans infection following radiation or chemoradiation (7), the possible role of the fungus in second primary tumor formation warrants further investigation.

## MATERIALS AND METHODS

### Ethics statement.

This study conformed with EU Directive 2010/63/EU and was approved by the regional Station for Animal Health and Food Control (Csongrád-Csanád, Hungary) under project license no. XXIX./4061/2020. The 4NQO model license was under United Kingdom Home Office license no. P292BBCE6.

### Cell lines and maintenance.

Two human oral squamous cell carcinoma (OSCC) cell lines (HSC-2 and HO-1-N-1) and an artificially immortalized telomerase-deficient oral epithelial cell line (OKF6/TERT2) were used. HSC-2 (JCRB0622) cells were cultured in Eagle's minimum essential medium (EMEM; Lonza), while HO-1-N-1 (JCRB0831) cells were cultured in Dulbecco's modified Eagle medium–F-12 medium (DMEM/F-12; Lonza), both containing 10% heat-inactivated (56°C, 30 min) fetal bovine serum (FBS) (EuroClone) supplemented with 4 mM glutamine, 100 U/mL penicillin, and 100 mg/mL streptomycin. OKF6/TERT2 cells were cultured in keratinocyte serum-free medium (KSFM) supplemented with 25 μg/mL bovine pituitary extract (BPE), 2 ng/mL recombinant epidermal growth factor (rEGF), 2 mM l-glutamine, 100 U/mL penicillin, and 100 mg/mL streptomycin. Cells were maintained at 37°C in the presence of 5% CO_2_.

### Wound healing assay to assess cellular migration.

HSC-2 and HO-1-N-1 cells were seeded at 3 × 10^5^ cells/well in 6-well plates per well. Cells were allowed to grow until 100% confluence. Scratches were made across the confluent cells using a P100 pipette tip. Zymosan (10-μg/mL working concentration) or heat-killed (HI) (65°C, 2 h) or live C. albicans SC5314 (SZMC 1523) or C. parapsilosis CLIB 214 (SZMC 1560) was used as the fungal treatments. In the case of HI *Candida*, the multiplicity of infection (MOI, tumor cells to fungal cells) was 1:10. Images were taken at time point 0 h (immediately after treatment) and 24 h. Cell migration speed was analyzed using ImageJ software. The rapid hypha formation of live C. albicans cells did not allow a comprehensive analysis of the extent of cancer cell movement after 24 h. Thus, a 24-h time-lapse (CytoSMART) video was analyzed to examine the extent of tumor cell migration. For this experiment, 1 × 10^5^ tumor cells were seeded into 24-well plates using 500-μm culture inserts and grown until full confluence. On the following day, the insert was removed and live *Candida* cells (MOI, tumor cells to fungal cells; for C. albicans, MOI of 400:1; for C. parapsilosis, MOI of 1:4) were added and imaged by time-lapse video with CytoSMART Lux2.

### BrdU incorporation assay.

Cell proliferation activity was measured by a BrdU incorporation assay using a cell proliferation ELISA kit (Sigma-Aldrich). The wells of 96-well plates were seeded with 5,000 HSC-2 or HO-1-N-1 cells. The following day, cells were treated with zymosan (10 μg/mL), HI *Candida* (MOI, 1:10), live C. albicans (MOI, 400:1), or C. parapsilosis (MOI, 1:4) for 24 h, and then the BrdU assay was performed according to the manufacturer’s instructions. The experiment was performed in medium supplemented with 1% FBS, 4 mM glutamine, 100 U/mL penicillin, and 100 mg/mL streptomycin.

### Sample preparation for metabolic analysis.

For metabolomic analyses, 1.5 × 10^5^ HSC-2 and HO-1-N-1 tumor cells were seeded per well in 6-well plates. On the following day, cells were treated with zymosan (10 μg/mL) or HI *Candida* (MOI, 1:10) for 24 h. After removal of medium, tumor cells were washed with phosphate-buffered saline (PBS) and extracted by the addition of 500 μL ice-cold mixture of HPLC-grade methanol-water (4:6, vol/vol), and the remaining debris was removed by scraping (20-mm blade width). Cell lysates were transferred to microcentrifuge tubes and sonicated for 5 min at 23 kHz in an ice water bath. Sonicated samples were mixed for 15 s and centrifuged at 13,800 × *g* for 10 min at 4°C. The supernatants were transferred to HPLC vials and stored at −80°C.

Due to the rapid hyphal formation of the live C. albicans, for the live yeast conditions, HSC-2 and HO-1-N-1 cells were seeded in 6-well plates (1.5 × 10^5^/well) in 5 technical replicates. After 4 h, the completely attached cancer cells were treated with live C. albicans (MOI, 400:1) or C. parapsilosis (MOI, 1:4) for 24 h. After removal of the culture medium, the cells were washed immediately one time in 37°C Ringer’s solution and then two times in 37°C HPLC-grade distilled water. Metabolite extraction was performed by incubation of the samples in 500 μL HPLC-grade distilled water for 15 min, causing osmotic shock for the tumor cells, while the fungal cell wall remained intact according to the literature (78) and based on our measurement of the supernatants collected from the *Candida* cells incubated in distilled water for 15 min, in which no any metabolites were detected. Samples were then centrifuged at 13,800 × *g* for 10 min at 4°C, and the supernatants were transferred to HPLC vials and stored at −80°C.

### HPLC-HRMS analysis of metabolites.

The amounts of the intermediaries of glycolysis, TCA cycle, and amino acids were determined by high-performance liquid chromatography coupled with high-resolution mass spectrometry (HPLC-HRMS). The measurements were carried out on a Dionex UltiMate 3000 (Thermo Scientific) HPLC system coupled to a Q Exactive Plus (Thermo Scientific) HRMS, where eluent A was water and eluent B was methanol, both supplemented with 0.1% acetic acid. The applied gradient program was as follows on a Synergi Polar-RP (Phenomenex) 250- by 3-mm, 4-μm column for eluent B: 0 min, 20%; 2 min, 20%; 4 min, 30%; 6 min, 95%; 9 min, 95%; 9.5 min, 20%; and 15 min, 20%. The flow rate and injection volume were 0.2 mL/min and 5 μL, respectively, while the column and the autosampler were thermostated at 30°C and 4°C, respectively. The HRMS was operated with a heated electrospray ionization (HESI) source in the parallel reaction monitoring (PRM) acquisition mode with polarity switching. During the measurements, the spray voltages were 4 kV and 3 kV in the positive and negative ionization modes, respectively. The sheath gas was 30 arbitrary units, the auxiliary gas was 15 arbitrary units, the auxiliary gas heater temperature was 250°C, and the ion transfer capillary temperature was 250°C in both ionization modes. The isolation window was 0.4 *m/z*, and the resolution was 35,000 (at *m/z *200). The precursor mass, fragment ion mass, polarity, retention time, and fragmentation energy and the lower limit of determination (LLOQ) of the examined metabolites are detailed in Data Set S1N in the supplemental material. For quantitative determinations, seven-level calibration curves were used in the case of each metabolite, in the range of 5 to 5,000 ng/mL. The calibration standard solutions were created in methanol (MeOH)-H_2_O (4:6) solution with the same amount of internal standard (250 ng/mL) as in the tumor cell extracts. For the live *Candida* treatment conditions, calibration standard solutions were created in HPLC-grade distilled water. Finally, the concentration values were compared with those of the control samples.

### MMP enzymatic activity.

Matrix metalloproteinase (MMP) activity was measured using the MMP activity assay kit (Abcam; ab112146), in accordance with the manufacturer’s instructions. For the experiments, 3 × 10^5^ cells were seeded into T25 flasks. On the following day, cells were treated with zymosan (10 μg/mL), HI *Candida* (MOI, 1:10), live C. albicans (MOI, 400:1), or C. parapsilosis (MOI, 1:4) for 24 h in 4 mL serum-free medium. Next, the medium was collected and centrifuged for 5 min at 3,000 × *g*, and 4 mL of the supernatant was concentrated to approximately 200 μL by centrifugation for 25 min at 7,500 × *g* using a centrifugal filter (Amicon Ultra-4; UFC800324). The concentrated samples were adjusted to the same volume. The activities of the MMPs were measured with a fluorescence plate reader at excitation/emission wavelengths of 490/525 nm.

### RNA extraction for sequencing *in vitro* samples.

For RNA extraction, 1 × 10^5^ HSC-2 and HO-1-N-1 cells were seeded into 24-well plates with three technical replicates. After 12 h, the tumor cells were treated with zymosan (10 μg/mL), HI C. albicans (MOI, 1:10), HI C. parapsilosis (MOI, 1:10), live C. albicans (MOI, 25:1), or live C. parapsilosis (MOI, 1:4) for 12 h. After fungal treatment, mRNA was purified using the RNeasy Plus minikit (Qiagen) according to the manufacturer's protocol. RNA quality and quantity were analyzed using a Bioanalyzer instrument (Agilent). Library preparation and sequencing on a NovaSeq S4 platform was performed by Novogene.

### RNA sequencing.

The preparation of the mRNA sequencing library was done by external specialists at Novogene. Briefly, sequencing libraries were generated using the NEBNext Ultra RNA library prep kit from Illumina (NEB, USA) in accordance with the manufacturer’s recommendations. For the purification of mRNA samples, poly(T) oligonucleotide-attached magnetic beads were used, and then size-selected cDNAs were synthesized with the AMPure XP system (Beckman Coulter, Beverly, MA, USA).

### Transcriptome analysis.

RNA sequence files were also processed by Novogene; however, the analyses of *in vivo* samples were repeated using our in-house protocol. According to the Novogene analysis pipeline, raw sequence reads processed for quality via fastp, reads with adaptor contamination, and a high percentage (*N* > 10%) of misreads or reads with uncertain bases and low quality (phred < 20) were filtered out. Clean read files were aligned to the reference genome index (GRCh38) using HISAT2, with the parameters –dta –phred33. Read counts of known and novel genes were quantified as reads per kilobase of exon model per million mapped reads (RPKM) via Featurecounts. This step in the case of the *in vivo* samples was interchanged with the GenomicAlignments package, resulting in counts. Differential gene expression in logarithmic fold change (LFC) was then determined using the DeSeq2 tool. Objects with read counts lower than 1 ppm were filtered out. In the experimentally derived gene list, differentially expressed genes (DEGs) were assigned above the absolute value of LFC of >1 and the adjusted *P* value of <0.05. The false discovery rate was minimized using the Benjamini and Hochberg approach.

### Causal analyses.

We employed causal analysis methods included in ingenuity pathway analysis (IPA), including (i) upstream regulatory analysis (URA) to identify probable upstream regulators and (ii) causal network analysis (CNA) to observe connections between these above-mentioned regulatory molecules. *A priori*, an expression core analysis was run on the experimentally derived gene set to obtain a suitable input for these analyses, and predictions with a *P* value of overlap of <0.05 and a Z-score different than 0 were considered significant hits. We further specified that only experimentally proven or strongly predicted intermolecular relationships should be considered.

### cDNA synthesis and reverse transcription-PCR for validation sequencing data.

A total of 1 μg RNA was used for cDNA synthesis using a RevertAid first-strand cDNA synthesis kit (Thermo Scientific) according to the manufacturer’s instructions. Real-time PCR was carried out in a final volume of 20 μL using Maxima SYBR green/fluorescein qPCR (2×) master mix (Thermo Scientific). The reaction was performed in a C1000 thermal cycler (Bio-Rad) using the following reaction conditions: 95°C for 3 min, 95°C for 10 s, 60°C for 30 s, and 65°C for 5 s for 50 cycles. The fold change in mRNA expression was calculated by the threshold cycle (ΔΔ*C_T_*) method (real-time PCR applications guide; Bio-Rad) using the *B2m* housekeeping gene as an internal control.

### Establishment of a novel *in vivo* mouse xenograft model of OSCC and oral candidiasis.

Sic- to eight-week old female BALB/c mice were immunocompromised with cortisone acetate for subsequent injection with human HSC-2 cells. Cortisone acetate was suspended in sterile Ringer’s solution containing 0.05% Tween 80 (vol/vol) and administered daily *per os* at a concentration of 225 mg kg^−1 41^ in a total volume of 0.2 mL using a sterile gavage needle (38 mm × 22 gauge curved). Because of the immunosuppression, autoclaved rodent feed and bedding were used. Drinking water was supplemented with 1% 100 U/mL penicillin and 100 mg/mL streptomycin.

Tumor cell injection was performed on the second day. HSC-2 cells were washed twice with 1× PBS and trypsinized. Trypsin was then neutralized with complete growth medium, and the cell suspension was centrifuged at 400 × *g* for 5 min. After removal of the supernatant, the cell pellet was suspended in serum-free medium. Anesthesia was performed by administration of 40 mg/kg pentobarbital intraperitoneally (i.p.), and 1 × 10^6^ HSC-2 cells were injected into the apex of the tongue in a 50-μL volume containing 10% (vol/vol) Matrigel (Corning). Mice were continuously monitored until they recovered from anesthesia.

Oral candidiasis was induced on day 5 with minor modifications to the protocol described previously by Solis and Filler (41). Briefly, C. albicans (SC5314) cells were inoculated in 5 mL yeast extract-peptone-D-glucose (YPD) and cultured for 24 h with continuous shaking at 30°C overnight. Inoculation and culturing were performed 3 times. *Candida* cells were then washed 3 times in 1× PBS and suspended in Hanks balanced salt solution (HBSS) to a concentration of 1 × 10^9^ cells mL^−1^. The C. albicans suspension was placed in a 30°C water bath for 5 min. Calcium alginate swabs were placed in the suspension approximately 5 min prior to use. Anesthesia was performed by administration of 40 mg/kg pentobarbital i.p. Saturated calcium alginate swabs were placed under the tongue of each mouse for 75 min. The following day, cortisone acetate was administered i.p. to avoid damage in the mouth. No cortisone was given day 7 or 8 because cortisone acetate absorbs more slowly through i.p. injection, resulting in a prolonged immunosuppressive effect. The weight of the animals was monitored every day. For validation of oral candidiasis, the tongue was excised and homogenized for approximately 8 to 10 s in PBS with a tissue homogenizer. Homogenates were used for determination of fungal burdens by colony counting after plating serial dilutions on YPD agar plates per tissue. The CFU were counted after 48 h of incubation at 30°C and expressed as CFU/g tissue. For CFU analysis, 4 mice were applied from each group.

### Sample preparation for sequencing from *in vivo* samples.

On the last day (8th) of the *in vivo* experiment, the mice were sacrificed and their tongues were removed for subsequent analyses. Mouse tongue tissues were removed from the tumor with the help of a scalpel. Next, 30 mg tumor tissue was homogenized (Bioneer) in RLT buffer, and RNA was extracted with an RNeasy Plus minikit (Qiagen) according to the manufacturer's instructions. RNA quality and quantity were analyzed using a Bioanalyzer instrument (Agilent). For transcriptome analysis, 4 mice were used from each group.

### Histopathological analysis.

The mice were sacrificed, and their tongues were removed from the base by use of dissecting scissors and forceps. Whole tongues were fixed in 4% formalin and kept at room temperature until specimen analysis. Fixed tongues containing tumor were sectioned and stained with periodic acid-Schiff (PAS) and hematoxylin-eosin (H&E) stains using conventional staining methods. For E-cadherin staining, standard immunohistochemical procedures were applied using rabbit monoclonal primary antibody (dilution, 1:200; EP700Y; Cellmarque). For vimentin staining, rabbit monoclonal primary antibody (dilution, 1:300; SP20; Cellmarque) was used. For p63 staining, mouse monoclonal primary antibody (dilution, 1:100; DBR16.1; Hisztopatológia Kft., Pécs, Hungary) was used. Antigen retrieval was performed by using epitope retrieval (ER) 2 solution (Leica; pH 9). The slides were then incubated with anti-E-cadherin, anti-vimentin, or anti-p63 antibodies for 20 min. Staining was performed with a Leica Bond Max automatic staining system using bond polymer refine detection (Leica). The slides were mounted with coverslips and assessed by microscopic examination (BX51 Olympus or Zeiss Imager Z1). For histopathological analysis, 8 mice were used from each group.

### qPCR analysis of carcinogens in OKF6/TERT2 cell line.

OKF6/TERT2, a telomerase-deficient oral epithelial cell line derived from a healthy individual, was used for the experiment and maintained as described previously (79). A total of 3.5 × 10^5^ cells were seeded into 12-well plates with three technical replicates. After 12 h, the tumor cells were treated with live C. albicans and C. parapsilosis at an MOI of 200:1 (tumor cells to fungal cells) for 12 h. cDNA synthesis and qPCR were performed as mentioned above.

### 4NQO mouse model of C. albicans-driven oncogenesis.

BALB/c female mice were given 40 μg/mL 4NQO in drinking water for 8 weeks (made up fresh every week), followed by 1 week of tetracycline (TCN; antibiotic, 0.1%, also in drinking water). Mice were infected with C. albicans BWP17+CiP30 on week 10. To infect mice, mice were sedated (110 mg/kg ketamine and 8 mg/kg xylazine i.p.), and a calcium alginate urethral cotton swab soaked in 6 × 10^8^ C. albicans yeast cells/mL was placed under the tongue for 75 min. This was done three times in week 10 at 2-day intervals to establish infection. Mice were continued on maintenance doses of both TCN (0.01%) and low-dose C. albicans (6 × 10^4^ yeast cells/mL), delivered via drinking water *ad libitum*, for a further 10 weeks. At week 12 (2 weeks following C. albicans infection), 2 mice per group were culled for initial assessment. The remaining mice were euthanized at week 20, and the tongues were harvested and halved longitudinally for histopathological assessment (*n* = 6 for the 4NQO plus C. albicans group, *n* = 8 for the 4NQO-alone group).

### Histological assessment of dysplasia induced in 4NQO model.

The entire intraoral mucosa (dorsal tongue, ventral tongue, buccal mucosa, palate, and floor of mouth) was assessed for papillomas or dysplasia. Epithelial dysplasia was graded as mild, moderate, or severe by assessing architectural and cytological atypia criteria, including loss of basal nuclear polarity, expansion of the basal compartment, loss of intercellular cohesion, hyperchromasia, and increased nuclear-to-cytoplasm ratio. Aberrant stratification and maturation involving the basal third or two-thirds of the epithelium were graded as mild or moderate dysplasia, respectively. No foci of severe dysplasia were identified. Each mouse tongue was assessed and graded for atypical architectural and cytological criteria by a qualified histopathologist (normal = 0, papilloma = 1, mild dysplasia = 2, moderate dysplasia = 3), and the percentage score (sum of each group divided by the highest possible score) was calculated for each experimental group. Specimens containing dysplasia were then subjected to six PAS-stained step sections, followed by PAS staining.

### Western blot analysis of MMP1 and MMP10 protein.

For the experiments, 8 × 10^5^ HSC-2 cells were seeded into a 10-cm petri dish. On the following day, cells were treated with live C. albicans (MOI, 400:1 and 1,600:1) and C. parapsilosis (MOI, 1:4) for 24 h. The amounts of MMP1 and MMP10 proteins were examined by Western blot analysis. Cells were washed with PBS, scraped, and collected by centrifugation (900 × *g*, 10 min). Cell pellet was resuspended in lysis buffer containing 50 mM Tris, 2 mM EDTA, 50 mM NaCl, 0.5 mM dithiothreitol (DTT), and protease inhibitor cocktail (cOmplete; Roche). Cells were mechanically lysed by 5 cycles of freezing and thawing in liquid nitrogen. The samples were then centrifuged (16,000 × *g*, 10 min), and the supernatant, containing the proteins, was used for further investigations. The protein concentration was measured with Bradford reagent (Bio-Rad) according to the manufacturer’s instructions. Then, 30 μg total protein of each sample was boiled for 10 min with protein loading buffer (60 mM Tris [pH 6.8], 2% SDS, 10% glycerol, 5% β-mercaptoethanol, 0.002% bromophenol blue) and loaded onto a 10% SDS-polyacrylamide gel. After separation (120 V, 90 min), the proteins were transferred to a nitrocellulose membrane (200 mA, 90 min), followed by blocking with 5% bovine serum albumin (BSA; Sigma) diluted in Tris-buffered saline containing 0.005% Tween (TBS-T). To detect the amount of MMP1 and MMP10, anti-MMP1 (ab137332; rabbit polyclonal antibody, 1:3,000; Abcam) and anti-MMP10 (ab261733; rabbit polyclonal antibody, 1:1,000; Abcam) antibodies diluted in 1% BSA–TBS-T were used overnight. To ensure equal loading of the samples, a GAPDH loading control was applied (G8795; mouse monoclonal antibody, 1:3,000; Merck).

On the following day, the membranes were washed 3 times in TBS-T and incubated with horseradish peroxidase (HRP)-conjugated goat anti-rabbit (Dako) secondary antibody in a 1:6,000 dilution in the case of MMP1 and in a 1:2,000 dilution for the detection of MMP10. For GAPDH, rabbit anti-mouse (Dako) secondary antibody was applied in a 1:6,000 dilution. The chemiluminescent signals on the membranes were developed using ECL reagent (Millipore) according to the instructions of the manufacturer, and the signal was detected in a C-DiGit blot scanner (LI-COR).

### Statistical analysis.

Statistical analysis was performed using the GraphPad Prism 7 software. All experiments were performed at least three times. Each replicate was normalized to its own control value, when it was necessary, and then normalized data were statistically analyzed. Paired or unpaired *t* tests were used to determine statistical significance (see figure legends for details), and differences between groups were considered significant at *P* values of <0.05 (*, *P* ≤ 0.05; **, *P* ≤ 0.01; ***, *P* ≤ 0.001; ****, *P* ≤ 0.0001).

### Data availability.

Processed and raw expression data are available through the Gene Expression Omnibus (https://www.ncbi.nlm.nih.gov/geo/) under accession number GSE169278.
