# Supplementary material for: Candida albicans Enhances the Progression of Oral Squamous Cell Carcinoma In Vitro and In Vivo
Source: mBio. 2022 Jan 4;13(1):e03144-21. doi: 10.1128/mBio.03144-21 (PMC8725587; doi:10.1128/mBio.03144-21)
Supplement: FIG S1 [file mbio.03144-21-sf001.pdf]

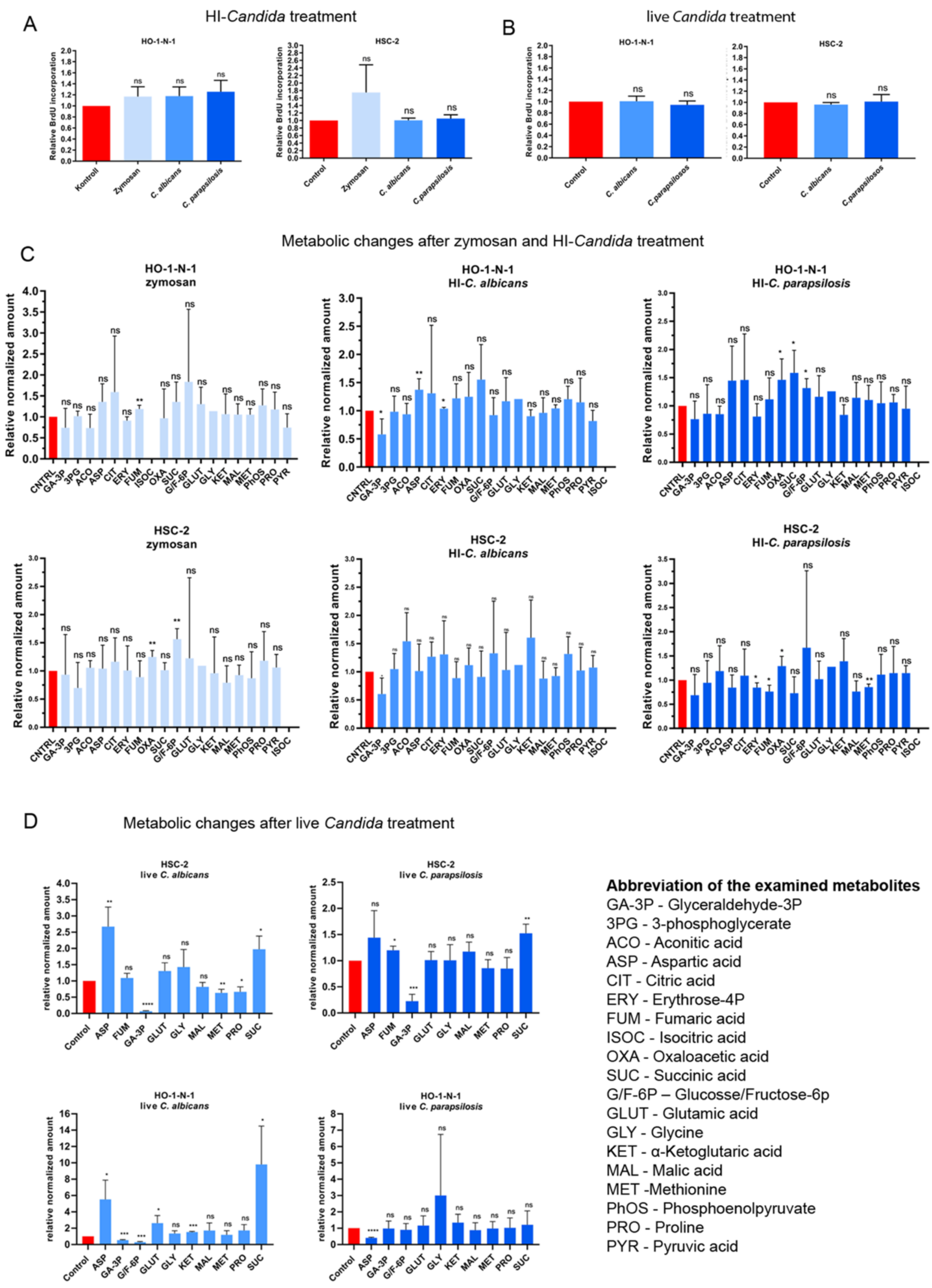

Supp Fig1

(A) Normalized proliferation activity of OSCC cells in the presence of heat- killed *C. albicans*, C. *parapsilosis* and zymosan measured by BrdU incorporation assay.

(B) Normalized proliferation activity of OSCC cells in the presence of live *C. albicans* and *C. parapsilosis* measured by BrdU incorporation assay.

(C) Normalized amount of metabolites of OSCC cells in the presence of heat- killed *C. albicans*, *C. parapsilosis* and zymosan measured by HPLC-HRMS.

(D) Normalized amount of metabolites of OSCC cells in the presence of live *C. albicans* and *C. parapsilosis* measured by HPLC-HRMS.

Unpaired t-test. * $p \leq 0.05$; ** $p \leq 0.01$, ***$p \leq 0.001$; **** $p \leq 0.0001$.
