# Supplementary material for: Candida albicans Enhances the Progression of Oral Squamous Cell Carcinoma In Vitro and In Vivo
Source: mBio. 2022 Jan 4;13(1):e03144-21. doi: 10.1128/mBio.03144-21 (PMC8725587; doi:10.1128/mBio.03144-21)
Supplement: FIG S2 [file mbio.03144-21-sf002.pdf]

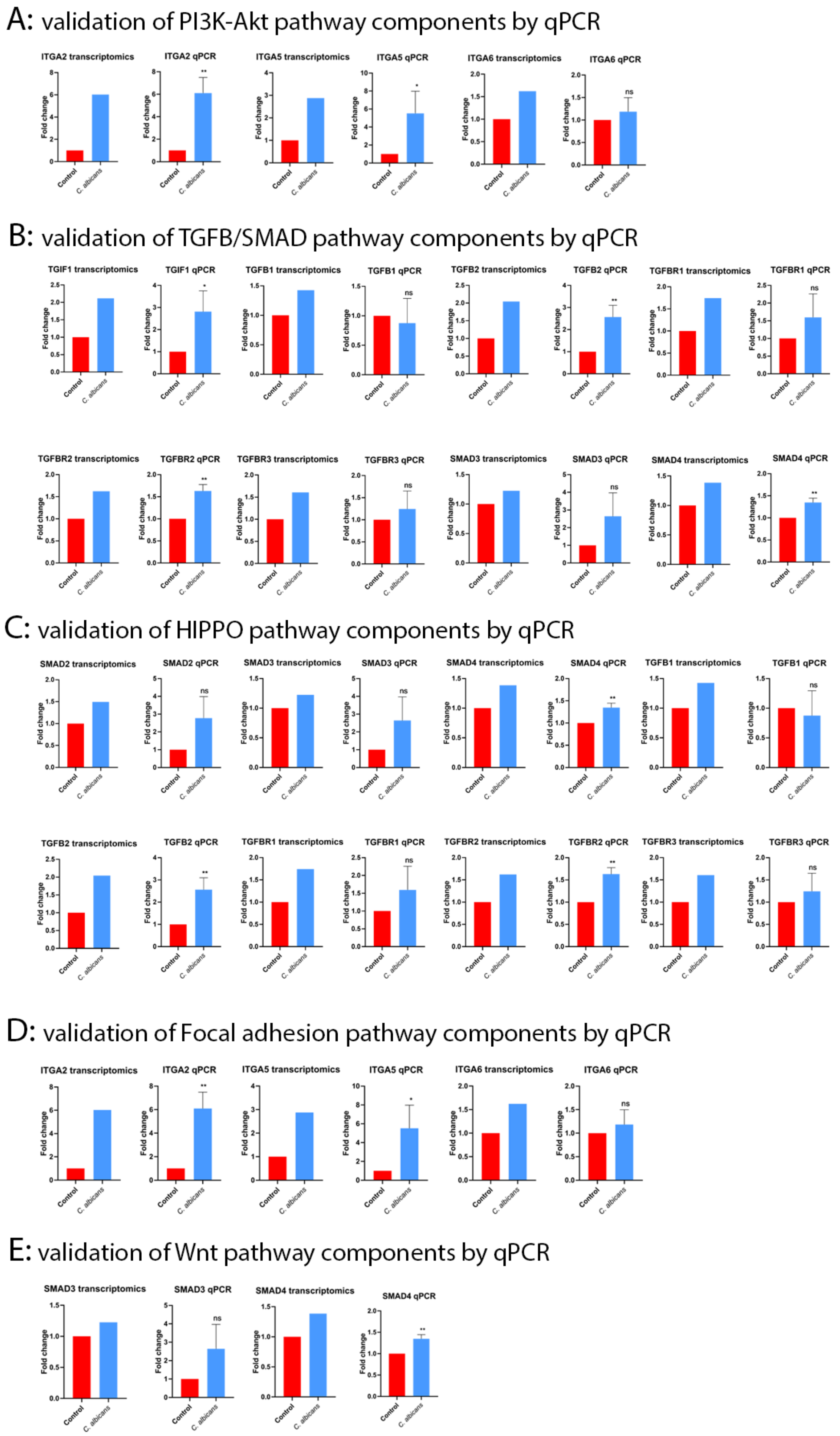

Supp Fig2

Validation of *C. albicans* activated signaling pathways. Validation was performed by qPCR analysis of pathway components.

(A) PI3K-Akt signaling pathway (B) TGF- β/SMAD (C) Hippo signaling pathway (D) Wnt signaling pathway (E) Focal adhesion pathway

Unpaired t-test. * $p \leq 0.05$; ** $p \leq 0.0$
