# Supplementary material for: Candida albicans Enhances the Progression of Oral Squamous Cell Carcinoma In Vitro and In Vivo
Source: mBio. 2022 Jan 4;13(1):e03144-21. doi: 10.1128/mBio.03144-21 (PMC8725587; doi:10.1128/mBio.03144-21)
Supplement: FIG S3 [file mbio.03144-21-sf003.pdf]

A

HSC-2 cell line *C. albicans* treatment in vitro; scSec data vs. *C. albicans* induced gene set

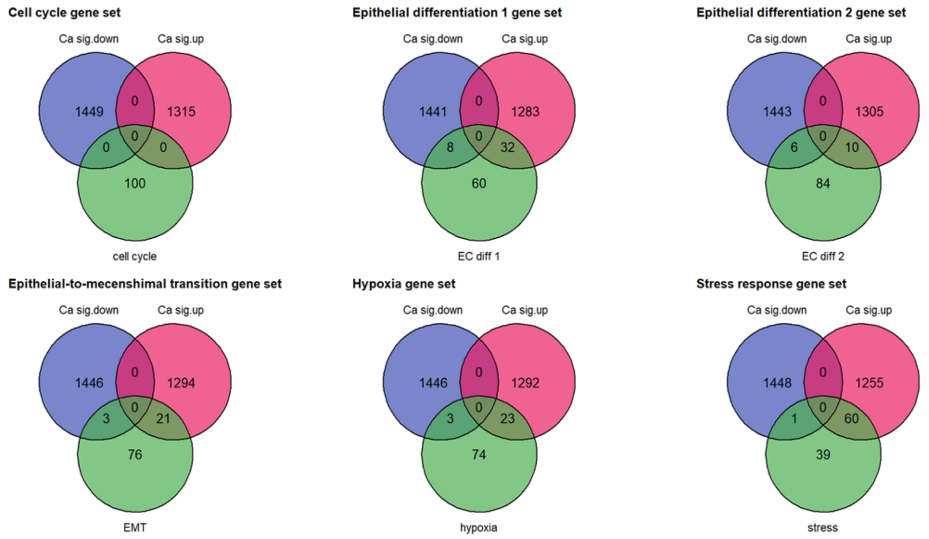

B

HO-1-N-1 cell line *C. albicans* treatment in vitro; scSec data vs. *C. albicans* induced gene set

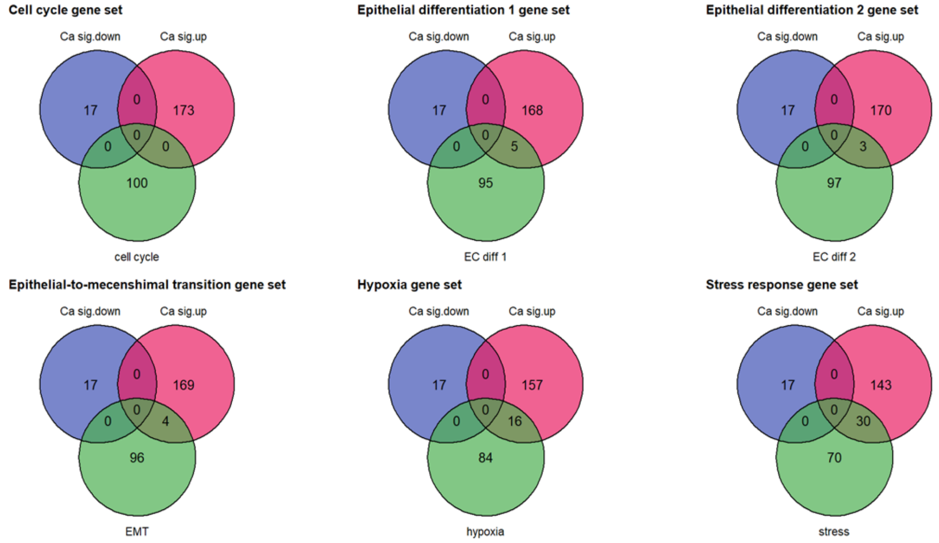

C

Live *Candida albicans* induced genes in both (HSC-2 and HO-1-N-1) cell lines, which are involved in OSCC progression

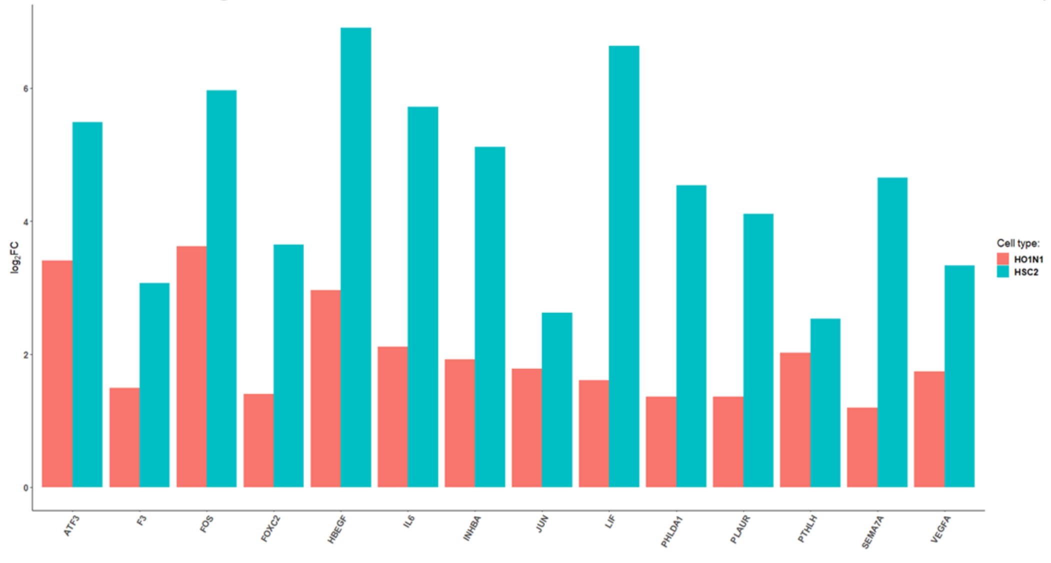

Supp Fig3

(A) Comparison of *Candida induced* genes in HSC-2 cell line to genes involved in different tumor progression processes. Differentially expressed gene list derived from an OSCC single cell sequencing study.

(B) Comparison of *Candida induced* genes in HO-1-N-1 cell line to genes involved in different tumor progression processes. Differentially expressed gene list derived from an OSCC single cell sequencing study.

(C) Live *C. albicans* induced genes in both (HSC-2 and HO-1-N-1) cell lines, which are involved in OSCC progression. OSCC progression marker gene list derived from literature and OSCC single cell sequencing study.
