# Supplementary material for: Candida albicans Enhances the Progression of Oral Squamous Cell Carcinoma In Vitro and In Vivo
Source: mBio. 2022 Jan 4;13(1):e03144-21. doi: 10.1128/mBio.03144-21 (PMC8725587; doi:10.1128/mBio.03144-21)
Supplement: FIG S4 [file mbio.03144-21-sf004.pdf]

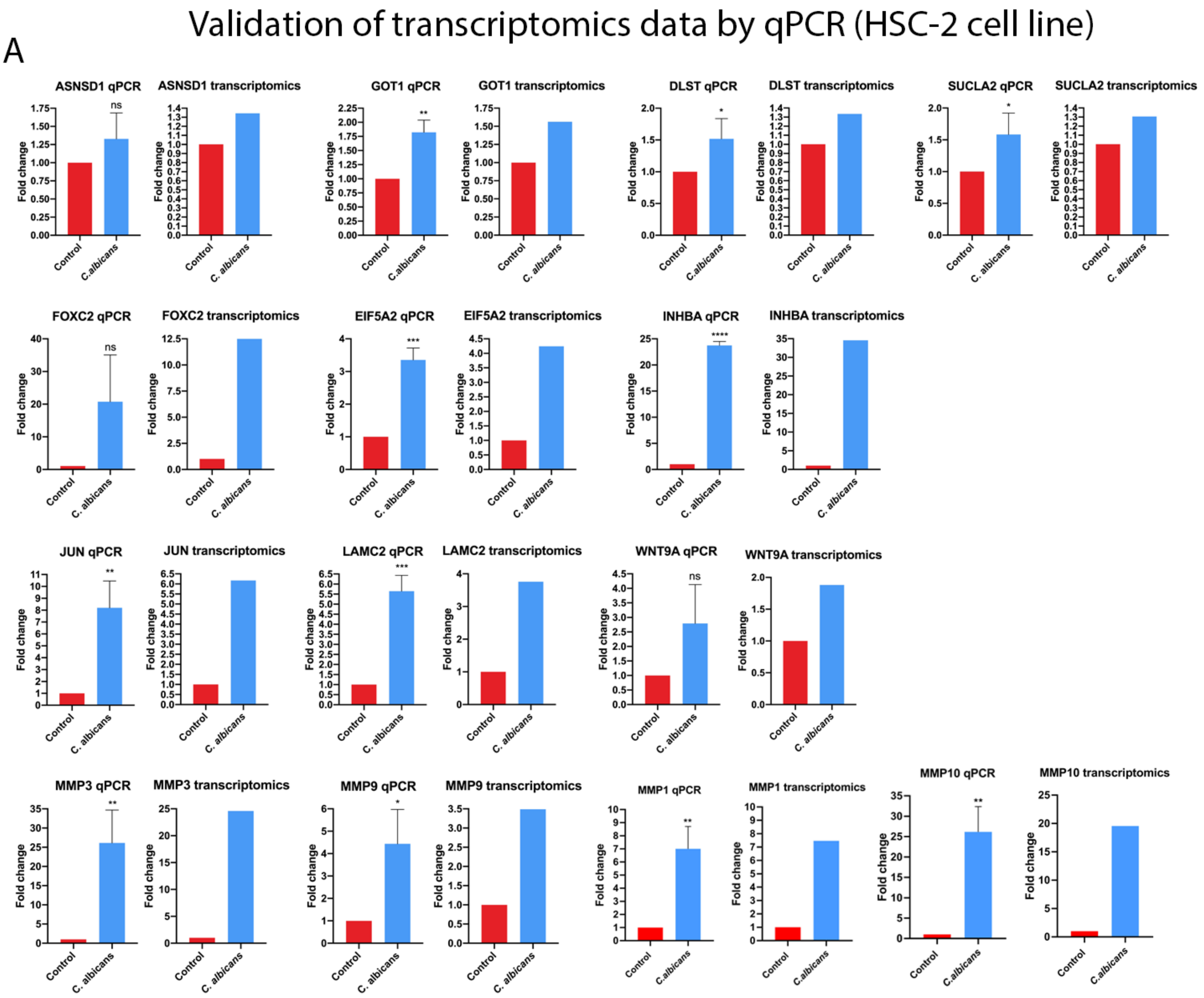

Supp Fig4

(A) Validation of transcriptomic data by qPCR

Unpaired t-test. * $p \leq 0.05$; ** $p \leq 0.01$, ***$p \leq 0.001$; **** $p \leq 0.0001$.
