# Supplementary material for: Candida albicans Enhances the Progression of Oral Squamous Cell Carcinoma In Vitro and In Vivo
Source: mBio. 2022 Jan 4;13(1):e03144-21. doi: 10.1128/mBio.03144-21 (PMC8725587; doi:10.1128/mBio.03144-21)
Supplement: FIG S5 [file mbio.03144-21-sf005.pdf]

A

	OSCC Xen.1	OC-OSCC Xen.1	OSCC Xen.2	OC-OSCC Xen.2	OSCC Xen.3	OC-OSCC Xen.3	OSCC Xen.4	OC-OSCC Xen.4	OSCC Xen.5	OC-OSCC Xen.5	OSCC Xen.6	OC-OSCC Xen.6	OSCC Xen.7	OC-OSCC Xen.7	OSCC Xen.8	OC-OSCC Xen.8
Inflammation in surface epithelium	0	1	0	3	0	2	0	2	0	3	0	3	0	3	0	2
Necrosis	0	1	1	1	1	1	1	1	0	1	0	1	0	0	0	0
Pushing (P) or invasive (I) tumor edge	I	P/I	P	I	I	I	P	I	I	P	I	P	P	I	P	P
Budding / EMT	1	1	2	1	1	2	1	2	2	2	1	2	1	3	1	2
Invasion	1	1	1	1	1	1	2	2	1	1	1	1	1	1	1	1
Thrombosis	0	1	1	2	0	1	0	0	0	0	0	0	0	1	0	1
Peritumoral inflammation	3g	1l/g	1l/g	1l/g	1l/g	1l/g	1l/g	2l/g	3g	1	1g	2	1g	3	2g	1

Inflammation in surface epithelium: 0 / 1 / 2 / 3
Necrosis : 0 / 1
Budding / EMT: 0 / 1 / 2
Invasion: 0 / 1 / 2 / 3
Thrombosis: 0 / 1
Peritumoral inflammation: 0 / 1 /2 /3
(g=granulocyte; l=lymphocyte)

normal/not detected = 0
mild difference = 1
moderate difference = 2
severe difference = 3

B

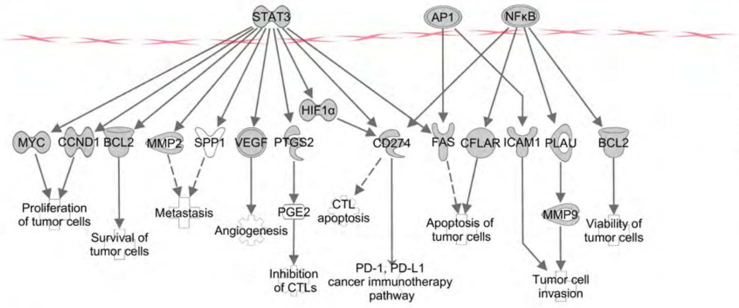

C

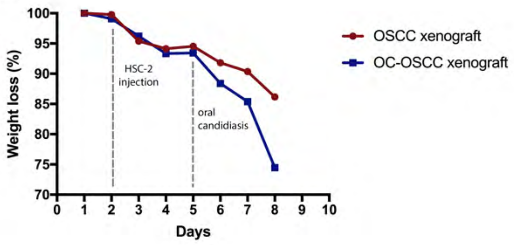

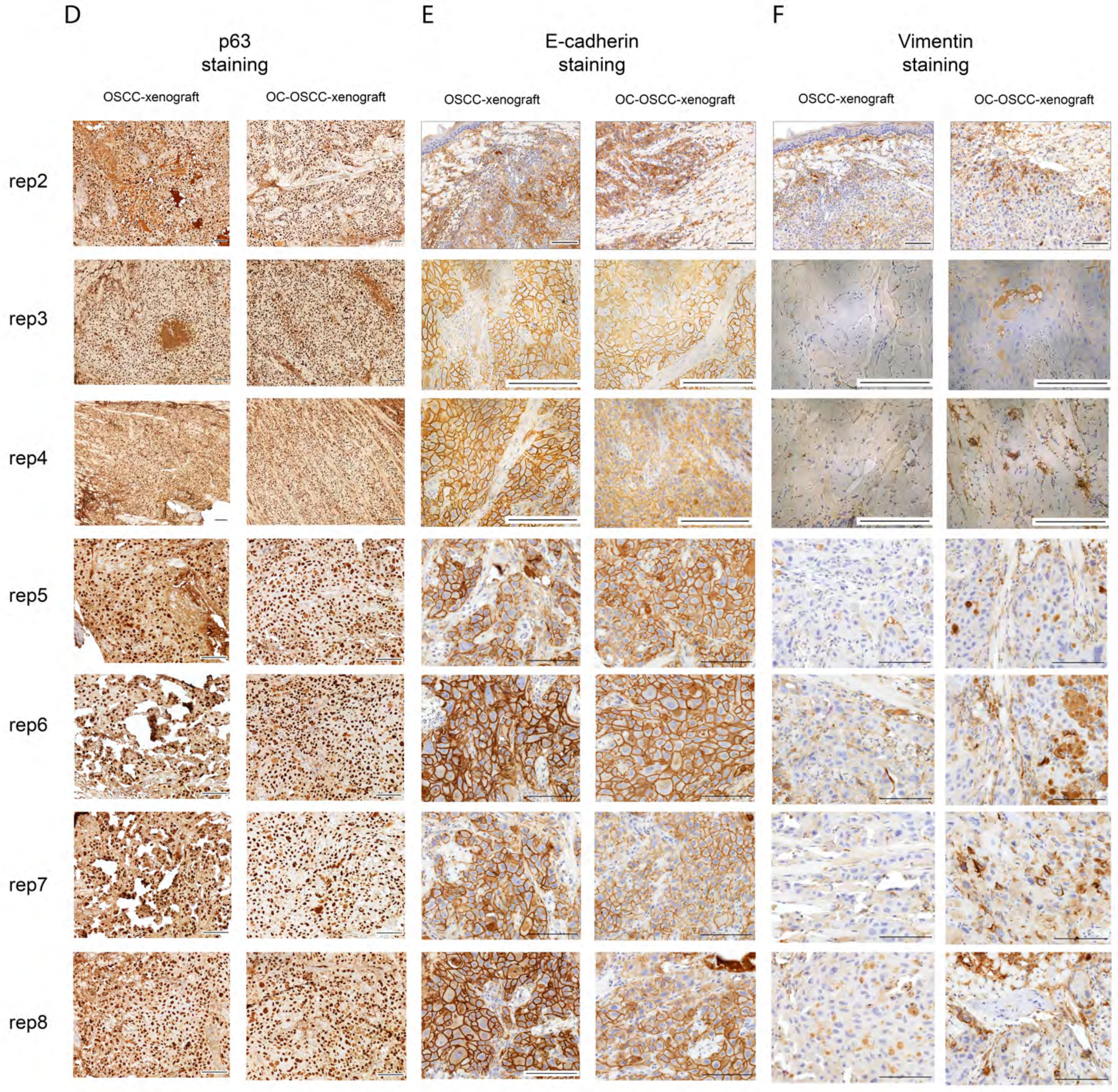

Supp Fig5

(A) Histopathological samples of *Candida*-colonized and *Candida*-free tumors, analysed and scored manually by a pathologist after H&E staining.

(B) Causal analyses of the genes which expression changed in OC-OSCC samples after oral candidiasis.

(C) Weight loss of the animals after HSC-2 tumor cell injection (OSCC xenograft) and HSC-2 injection combined with oral candidiasis (OC-OSCC xenograft).

(D) p63 staining of the histopathological samples (animal 2, 3, 4)

(E) E-cadherin staining of the histopathological samples (animal 2, 3, 4)

(F) vimentin staining of the histopathological samples (animal 2, 3, 4)
