# Supplementary material for: Candida albicans Enhances the Progression of Oral Squamous Cell Carcinoma In Vitro and In Vivo
Source: mBio. 2022 Jan 4;13(1):e03144-21. doi: 10.1128/mBio.03144-21 (PMC8725587; doi:10.1128/mBio.03144-21)
Supplement: FIG S6 [file mbio.03144-21-sf006.pdf]

A

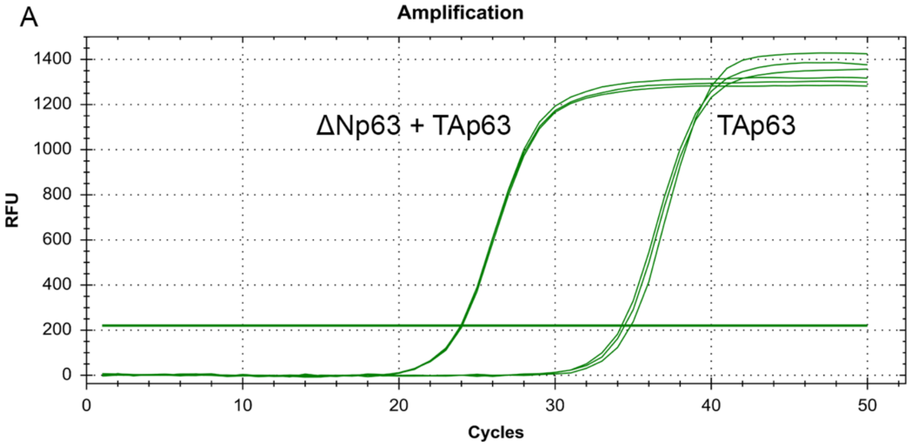

B

	qC
ΔNp63+TAp63	24.02
ΔNp63+TAp63	23.96
ΔNp63+TAp63	24.06
TAp63	34.45
TAp63	34.26
TAp63	34.85

Western blot analysis of MMP10 and MMP1 protein

C

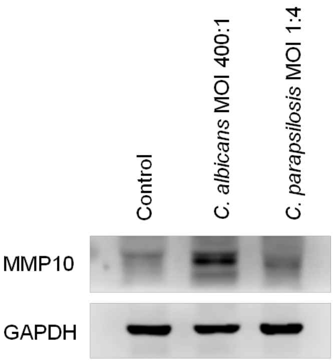

D

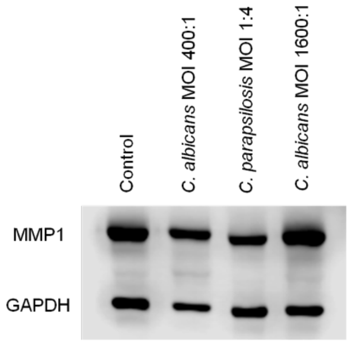

E

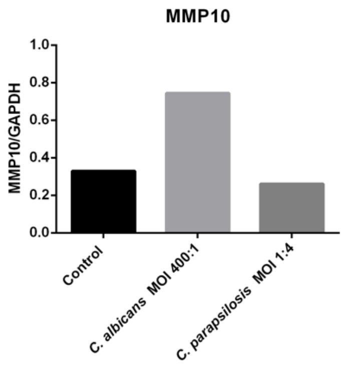

F

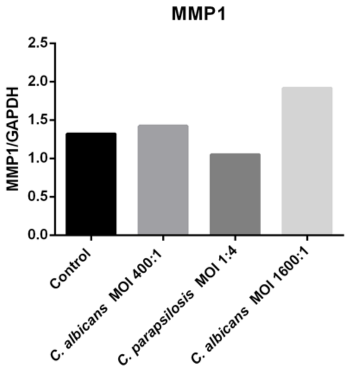

Supp Fig6

(A) qPCR curve of TAp63 and ΔNp63+TAp63 splice variants. First primer pair was designed close to C-terminal region, second primer pair in the N-terminal region. First primer pair amplifies both splice variant group (ΔNp63+TAp63), secon primer pair amplifies only splice variant posessing N-terminal region (TAp63).

(B) qC value of the transcript variants.

(C) Western blot analysis of MMP10 protein (MOI: tumor cell: fungal cell)

(D) Western blot analysis of MMP1 protein (MOI: tumor cell: fungal cell)

(E) Densitometry data generated from MMP10 Western blot results

(F) Densitometry data generated from MMP1 Western blot results
